# Protective human antibodies against a conserved epitope in pre- and postfusion influenza hemagglutinin

**DOI:** 10.1073/pnas.2316964120

**Published:** 2023-12-26

**Authors:** Joel Finney, Annie Park Moseman, Susan Kong, Akiko Watanabe, Shengli Song, Richard M. Walsh, Masayuki Kuraoka, Ryutaro Kotaki, E. Ashley Moseman, Kevin R. McCarthy, Dongmei Liao, Xiaoe Liang, Xiaoyan Nie, Olivia Lavidor, Richard Abbott, Stephen C. Harrison, Garnett Kelsoe

**Affiliations:** ^a^Laboratory of Molecular Medicine, Children’s Hospital, Harvard Medical School, Boston, MA 02115; ^b^Department of Integrative Immunobiology, Duke University, Durham, NC 27710; ^c^Department of Surgery, Duke University, Durham, NC 27710; ^d^The Harvard Cryo-Electron Microscopy (Cryo-EM) Center for Structural Biology, Harvard Medical School, Boston, MA 02115; ^e^Department of Biological Chemistry and Molecular Pharmacology, Blavatnik Institute, Harvard Medical School, Boston, MA 02115; ^f^Center for Vaccine Research, University of Pittsburgh School of Medicine, Pittsburgh, PA 15261; ^g^HHMI, Boston, MA 02115; ^h^Duke Human Vaccine Institute, Duke University, Durham, NC 27710

**Keywords:** memory B cell, ADCC, influenza vaccines

## Abstract

Phylogenetically and antigenically distinct influenza A and B viruses (IAV and IBV) circulate in human populations, causing widespread morbidity. Antibodies (Abs) that bind epitopes conserved in both IAV and IBV hemagglutinins (HAs) could protect against disease by diverse virus subtypes. Only one reported HA Ab, isolated from a combinatorial display library, protects against both IAV and IBV. Thus, there has been so far no information on the likelihood of finding naturally occurring human Abs that bind HAs of diverse IAV subtypes and IBV lineages. We have now recovered from several unrelated human donors five clonal Abs that bind a conserved epitope preferentially exposed in the postfusion conformation of IAV and IVB HA2. These Abs lack neutralizing activity in vitro but in mice provide strong, IgG subtype–dependent protection against lethal IAV and IBV infections. Strategies to elicit similar Abs routinely might contribute to more effective influenza vaccines.

## Significance

Current strategies for influenza virus vaccination rely on predictions about which viral strains will predominate during the coming flu season. Mismatches between the predicted and circulating strains often lower the effectiveness of the seasonal vaccine. A better vaccine would focus the immune response on viral structures conserved in most flu strains. We identified in several unrelated human subjects a group of Abs that bind a previously uncharacterized epitope conserved in all influenza virus subtypes currently circulating in the human population, as well as many subtypes that circulate in other animal populations and have the potential to cause human pandemics. These Abs protect mice against lethal flu infections. Vaccines that elicit similar Abs might be more effective than the current formulations.

Influenza A and B viruses (IAVs and IBVs) comprise multiple phylogenetically and antigenically distinct groups, of which two A lineages (H1N1 and H3N2) and two B lineages (Victoria and Yamagata) currently circulate among humans (1). Antibody (Ab) responses to IAV/IBV infection or vaccination are largely directed toward the viral hemagglutinin (HA) glycoprotein, which mediates viral attachment and membrane fusion with the host cell (1). Population-level immune pressure drives selection of HA mutations that promote viral escape from immune control (2, 3). Consequently, annually updated, licensed influenza vaccines typically protect only against seasonal strains and closely related subtypes (4, 5).

The HA trimer exposes its three, independently folded, receptor-binding “head” domains at the membrane-distal end of an elongated “stem.” Mutations in the immunodominant head are responsible for annual antigenic drift. The absence of immune pressure probably accounts for conservation of the stem, which is sequestered by tight HA packing on the virion surface from exposure to B cell receptors and Abs (6). Functional requirements for fusion may also limit mutational possibilities in the stem. Abs to conserved stem epitopes, as well as those to an epitopic region on the head interface or to the receptor site, protect against infection or disease by diverse subtypes and lineages of IAVs or IBVs (7–10), but such broadly protective HA Abs are rare. The only example of a protective monoclonal Ab (mAb) that cross-reacts with both IAV and IBV HAs is CR9114, a stem Ab isolated from a phage-displayed combinatorial Ab library (7). Human Abs with naturally paired heavy (H)- and light (L)-chains that protect against both IAV and IBV infection or disease have yet to be detected.

The presence of neutralizing Abs—as measured by hemagglutination inhibition (HAI) titer—is the only commonly accepted correlate of protection against influenza infection (11). Accordingly, research on IAV/IBV vaccines has focused largely on eliciting Abs with HAI activity and/or those that directly block viral entry (12–14). Although an Ab response that prevents infection is strictly necessary for prophylaxis, even non-neutralizing Abs can effectively mediate protection against morbidity by promoting clearance of virus and infected cells, through complement-dependent cytotoxicity (CDC), Ab-dependent cellular phagocytosis (ADCP), and Ab-dependent cellular cytotoxicity (ADCC). Indeed, these mechanisms are the principal means by which many broadly reactive HA Abs confer protection against lethal infection (10, 15, 16). Examples of protective, CDC/ADCP/ADCC-dependent Abs include those that bind HA in its prefusion conformation (10, 15–17) as well as those that preferentially bind HA in its postfusion state (18–21). The relevant epitopes of the latter Abs are probably occluded in the prefusion HA structure, but exposed in the postfusion structure. Alternatively, some epitopes may be absent in the prefusion conformation, but later generated by the fusogenic conformational rearrangement.

We report here identification and characterization of B cells, from four unrelated individuals, secreting IgGs that bind cell-surface expressed HA from various IAV subtypes and from both IBV lineages. The IgGs bind the corresponding postfusion HA2s in vitro, as well as the recombinant, soluble, HA0 trimeric ectodomains in Luminex and enzyme-linked immunosorbent assays (ELISAs). The structure of a Fab from one of the IgGs (S1V2-72) bound with the “EHA2” recombinant mimic (22) of postfusion HA2 defines the epitope as a β-hairpin loop; this β-hairpin is also present on the prefusion HA trimer, but it is occluded at the membrane-proximal end of the molecule. The fusogenic conformational change translocates this loop to the membrane-distal end of the postfusion form. S1V2-72 IgG does not neutralize infectivity in vitro but confers Fc-dependent protection from lethal challenge by either an IAV or an IBV.

## Results

### IAV and IBV Cross-Reactive HA Abs.

**Fig. 1.**
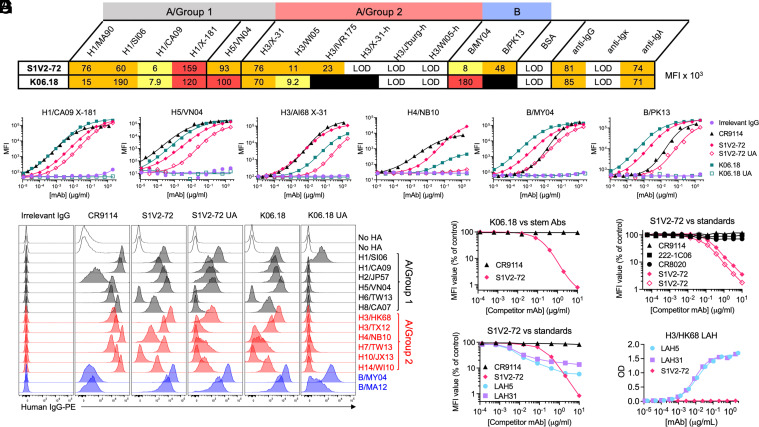
S1V2-72 and K06.18 are broadly binding HA stem mAbs whose epitope is distinct from those of other standard stem mAbs. (*A*) Results of a Luminex multiplex binding assay conducted with culture supernatant IgG from human Bmem clones S1V2-72 and K06.18. Binding mean fluorescence intensities (MFIs) are color-coded by magnitude: yellow (≥1 × 10^3^), orange (≥1 × 10^4^), red (≥1 × 10^5^), black: not done. LOD: below limit of detection. (*B*) Serially diluted recombinant IgG forms of S1V2-72, K06.18, their UAs, CR9114, and an irrelevant mAb were screened by Luminex assay for binding to full-length, trimeric, soluble prefusion rHA ectodomains (FLsE). (*C*) Flow cytometry histograms depicting recombinant IgG binding to K530 cell lines expressing recombinant, native HA on the cell surface. (*D*–*F*) Ab epitope overlap determined by competitive inhibition of binding in a Luminex assay. Each curve depicts the binding MFI of K06.18 (*D*) or S1V2-72 (*E* and *F*) to HA-conjugated microbeads in the presence of serially diluted competitor mAbs. HAs used: H1/X-181 (*D*), H1/CA09 (*E*; for CR9114, 222-1C06, and S1V2-72 filled diamonds) or H3/X-31 (*E*; for CR8020 and S1V2-72 empty diamonds), or H5/VN04 (*F*). (*G*) ELISA measurement of recombinant IgG binding to a truncated peptide encompassing the LAH of prefusion HA2. LAH5 and LAH31 are mAbs reported (18, 21) to bind the LAH.

To identify IAV- and IBV-cross-reactive HA Abs, we cultured individual HA-binding IgG^+^ memory B (Bmem) cells sorted from peripheral blood mononuclear cells (PBMCs) of eight human donors before or 1 to 2 wk after seasonal influenza vaccination (*SI Appendix*, Fig. S1*A*) (10, 23). We then used a multiplex binding assay to screen the secreted clonal IgGs for reactivity against a panel of recombinant HAs (rHAs; *SI Appendix*, Fig. S1*B* and Table S1); results from some of these cultures have been reported (10, 23). From eight donors, we recovered 1,642 IgG^+^ HA-binding cultures and identified two clonal Abs, S1V2-72 and K06.18, from unrelated donors, that bound rHAs from group 1 (H1 and H5) and group 2 (H3) IAV strains, as well as from the Victoria and Yamagata lineages of IBV (Fig. 1*A*). We identified the K06.18 Ab in an earlier report but did not characterize it (23).

S1V2-72 is encoded by IGHV1-2*02/IGHD5-12*01/IGHJ5*02 and IGLV2-23*01/IGLJ3*02, with 10.5% and 3.8% somatic mutations in the heavy (H) and light (L) chain variable genes, respectively. K06.18 is encoded by similar *V(D)J* rearrangements (IGHV1-2*07/IGHD6-13*01/IGHJ4*02 and IGLV2-23*02/IGLJ3*02) and is also substantially mutated in the H- (6.1%) and L-chain (5.4%) variable regions (23). No other Bmem cells clonally related to S1V2-72 or K06.18 were recovered from donors S1 or KEL06.

Recombinant S1V2-72 and K06.18 bound H1 HAs spanning ≥41 y of antigenic evolution, H3 HAs spanning ≥49 y, H4/NB10, H5/VN04, and HAs from the Yamagata and Victoria lineages of IBV (Fig. 1*B* and *SI Appendix*, Fig. S1 *C–E*). The inferred unmutated ancestor (UA) (24) of S1V2-72 also bound many of these HAs with substantial avidity, whereas the K06.18 UA did not bind any of the rHAs screened by our Luminex assays. In addition to binding bead-conjugated rHAs, S1V2-72 and K06.18 rAbs bound IAV and IBV rHAs expressed on the surface of the K530 cell line (Fig. 1*C*) (25). The S1V2-72 UA and K06.18 UA Abs also bound cell-surface rHAs, although less avidly than their mutated descendants, implying that selection for enhanced binding to HA encountered by infection or vaccination drove affinity maturation of S1V2-72 and K06.18. That the K06.18 UA Ab bound to some cell-surface HAs, but not to the corresponding HAs conjugated to Luminex beads, suggests that cell-surface display better exposes the relevant epitope.

S1V2-72 and K06.18 bound full-length HA trimers, but not “head-only” trimer constructs lacking the HA stem region (Fig. 1 *A* and *B* and *SI Appendix*, Fig. S1*F*) (10), implying that these Abs engage stem epitopes. Competitive binding assays showed that S1V2-72 strongly inhibits K06.18 binding (Fig. 1*D*), indicating that the two Abs recognize the same or overlapping epitopes. S1V2-72 did not, however, compete with standard Abs that define known conserved HA stem epitopes, including CR9114 (7), CR8020 (26), and 222-1C06 (27) (Fig. 1*E*). We confirmed that the S1V2-72/K06.18 epitope is distinct from those previously characterized by mutating conserved head and stem epitopes in rH3/KS17 to abolish the binding of epitope-defining standard Abs (28); no defining set of mutations strongly affected S1V2-72 reactivity (*SI Appendix*, Fig. S1*G*).

We note that S1V2-72 competed modestly with LAH5 and LAH31 (Fig. 1*F*), two cross-reactive mAbs with epitopes contained in the long α helix of the HA2 subunit (18, 21). LAH5 and LAH31 bound avidly to an HA peptide comprising solely the long α helix of prefusion HA2, but S1V2-72 did not (Fig. 1*G*). Therefore, the epitope of S1V2-72 must be distinct from those of the long α helix mAbs.

### Cryo-EM Structure of S1V2-72 in Complex with Postfusion HA2.

We were unable to isolate complexes of S1V2-72 Fab bound to trimeric B/MY04 HA0 ectodomain. Nonetheless, we infer that the S1V2-72 epitope must be at least partially exposed in native HA0 because S1V2-72 IgG bound HA0s conjugated to Luminex beads (Fig. 1*B* and *SI Appendix*, Fig. S1 *C–E*) and because S1V2-72 IgG immobilized on a biolayer interferometry (BLI) sensor captured soluble trimers of B/MY04 HA0 (*SI Appendix*, Fig. S2*A*). In the converse experiment, however, HA0 immobilized on the sensor did not capture S1V2-72 Fab (*SI Appendix*, Fig. S2*B*). Thus, although multivalent S1V2-72 Ig binds HA0, monovalent S1V2-72 Fab apparently binds HA0 too weakly for detectable complex formation. S1V2-72 Fab avidly bound B/MY04 EHA2, a postfusion HA2 construct (*SI Appendix*, Fig. S2*C*) (22), and we used the resulting complex for single-particle cryoelectron microscopy analysis.

**Fig. 2.**
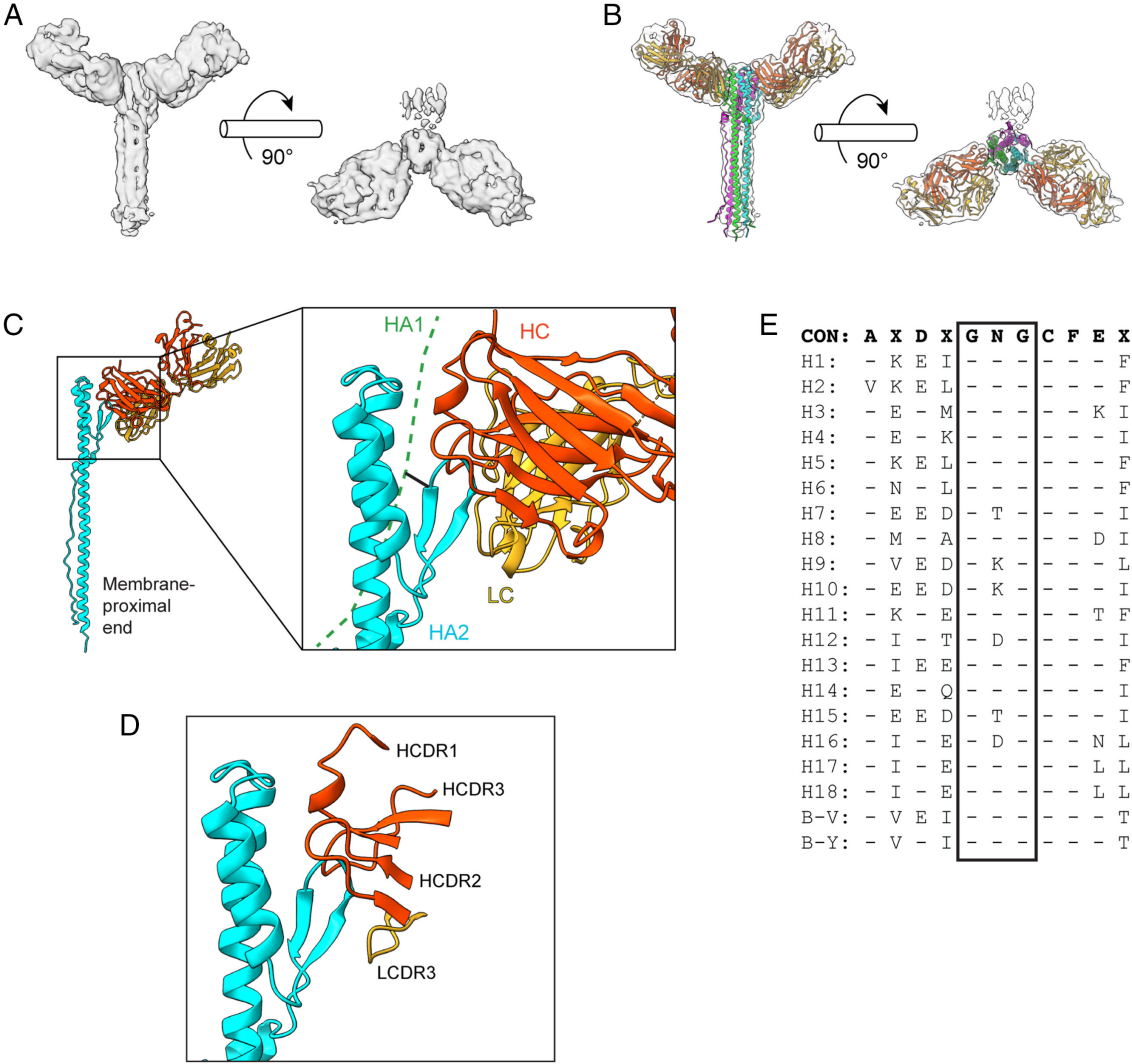
S1V2-72 binds a conserved β-hairpin exposed in EHA2. (*A*) 5Å-resolution cryo-EM map for S1V2-72 Fab bound to B/MY04 EHA2. (*B*) Docked model showing the AF2-predicted structures (ribbon models) of two S1V2-72 Fabs and trimeric B/MY04 EHA2 docked into the cryo-EM map shown in (*A*). (*C*) *Left*: ribbon model showing S1V2-72 Fab (heavy chain in orange; light chain in yellow) complexed with B/MY04 EHA2 monomer (cyan). *Right*: Rotated, close-up view of the S1V2-72:EHA2 interface. Also shown is the position of the HA1 strand (dashed green line) that would be linked by disulfide bond (black line) to HA2 in the postfusion structure of authentic HA1+HA2. (*D*) A simplified close-up view of the S1V2-72:EHA2 interface, showing only EHA2, the HCDRs, and LCDR3. (*E*) Alignment of the amino acid sequences encoding the β-hairpin in different HA subtypes. The boxed residues form the loop of the hairpin. Dashed positions indicate identity with the consensus residue. CON: consensus sequence. B-V: Victoria lineage of IBV. B-Y: Yamagata lineage of IBV.

We recorded 14,357 images with a Titan Krios electron microscope and a Gatan K3 detector. Two-dimensional class averages from a total of ~6,780,000 particles, calculated in CryoSPARC (29), showed that most of the complexes had shed one or more of their bound Fabs (*SI Appendix*, Figs. S3 and S4 *A* and *B*). We selected classes corresponding to both one bound Fab and two bound Fab; the latter refined more readily in three dimensions than did the former, and we chose the corresponding three-dimensional reconstruction (from about 60,000 contributing particles) as a reference for further refinement in CryoSPARC, to a final resolution of about 5Å (Fig. 2*A* and *SI Appendix*, Figs. S3 and S4 and Table S2).

We used AlphaFold2 (AF2) (30) to prepare a model of EHA2 with the sequence of the B/Malaysia/2506/2004 HA2 ectodomain (residues 23 to 181, H3 numbering) in its postfusion conformation. We also used AF2 to model the variable module of S1V2-72 and compared this model with the structure of the inferred germline Fab from the DH270 lineage (31), which derives from precisely the same VH and VL as S1V2-72. The AF2 and DH270 variable modules were essentially identical, except for the HCDR3, which is 20 residues in DH270 but only 12 in S1V2-72. Independent docking of the three modules (EHA2 and variable modules from AF2, plus the constant-region dimers from the DH270 UCA) produced an excellent fit to the map (Fig. 2*B*).

The center of the epitope is a β-hairpin, residues 129 to 141, at the membrane-distal end of the postfusion HA2 rod (Fig. 2*C*). In the Fab complex, the tip of the hairpin fits into a shallow cavity lined by the three heavy-chain CDRs, with additional contact from the light-chain CDR3 (Fig. 2*D*). The apparent interactions outside the β-hairpin are between the tips of both HCDR1 and HCDR2 and residues around the position at which the HA2 polypeptide chain folds back on itself when the extended intermediate collapses into the postfusion conformation (Fig. 2*D*). The flexibility of the hairpin in the recombinant EHA2 construct (22) precludes more precise modeling at this stage.

In the prefusion HA trimer, the β-hairpin is part of a small, five-strand sheet, which fixes its conformation more firmly than in EHA2. The central strand of that sheet comprises residues from HA1, including Cys14, which forms a disulfide link with HA2 Cys137 in the flanking strand—the descending strand of the β-hairpin epitope. The full postfusion structure thus retains HA1, flexibly tethered to the HA2 hairpin through that disulfide. Our fit to the density map suggests that the interaction of S1V2-72 with the β-hairpin can accommodate that tether (Fig. 2*C*). The hairpin's conformation in the prefusion structure is nearly the same as its average conformation in postfusion HA2, but the trimer interface in prefusion HA would prevent Ab access. Nonetheless, conformational fluctuations at the membrane-proximal end of prefusion HA might expose that loop, allowing transient access. Our Luminex and BLI data (Fig. 1 *A* and *B* and *SI Appendix*, Figs. S1 *C*–*E* and S2*A*) suggest that such fluctuations may indeed occur, at least in the context of the soluble, trimeric ectodomain.

The breadth of S1V2-72 binding can probably be attributed in part to the conserved primary structure of the β-hairpin (Fig. 2*E*). The bend of the hairpin encompasses Gly134-Asn135-Gly136, a sequence conserved in HAs from both lineages of IBV, plus all IAV HA subtypes except H7, H9, H10, H12, H15, and H16. The descending β strand continues with the absolutely conserved pair of Cys137 (and its disulfide linkage to Cys14 in HA1) and Phe138. Further study is needed to determine whether the amino acid substitutions in H9, H12, H15, and H16 prohibit S1V2-72 binding. S1V2-72 accommodates substitution of Thr for Asn135 in H7 (Fig. 1*C*), whereas substitution of Lys for Asn135 in H10 might account for its lack of S1V2-72 binding (Fig. 1*C*).

### Protection by S1V2-72.

**Fig. 3.**
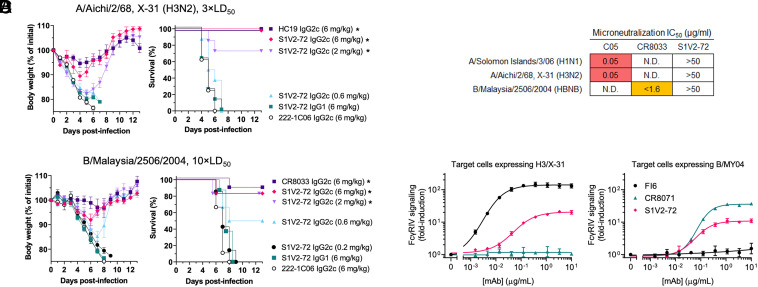
S1V2-72 protects against lethal IAV or IAB challenge by IgG subtype–dependent mechanisms. (*A* and *B*) Body weight and survival of mAb-infused mice after infection with H3N2 (*A*) or HBNB (*B*) influenza virus. Asterisks denote significant differences (*P* < 0.05) in survival at day 13 compared to 222-1C06-treated controls. (*C*) In vitro microneutralization IC_50_ values. (*D*) Results of an in vitro ADCC proxy assay. Mouse FcγRIV-expressing effector cells and HA-expressing target cells were cocultured in the presence of serially diluted IgG2c mAbs. FcγRIV activation was measured as luminescence output, as described in *Materials and Methods*.

To determine whether S1V2-72 can protect against lethal IAV or IBV infections, we passively transferred to mice the following IgGs: S1V2-72, the potent neutralizing HA Abs HC19 (32) or CR8033 (7), or an H1-specific Ab (222-1C06) (27). When administered as IgG2c, each mAb was injected at 6 mg Ab/kg mouse; alternatively, S1V2-72 IgG2c was transferred at doses lower than 6 mg/kg or as S1V2-72 IgG1 (at 6 mg/kg). Three hours after mAb infusion, mice were infected intranasally with H3N2 IAV (A/Aichi/2/1968, X-31) or IBV (B/Malaysia/2506/2004). All animals treated with the irrelevant 222-1C06 IgG2c lost 20% of their body weight by 4 to 8 d postinfection, requiring ethical euthanasia according to the protocol approved by the Duke University Institutional Animal Care and Use Committee (Fig. 3 *A* and *B*). In contrast, H3N2- and IBV-neutralizing Abs were potently protective. S1V2-72 protected efficiently against infection-induced weight loss when administered as mouse IgG2c, but failed to protect when administered as mouse IgG1 (Fig. 3 *A* and *B* and *SI Appendix*, Fig. S5*A*). In an in vitro influenza microneutralization assay, S1V2-72 neutralized neither IAV nor IBV, in contrast to receptor binding site Abs C05 (8) and CR8033 (Fig. 3*C*). As an IgG2c, S1V2-72 induced FcγRIV signaling in an Ab-dependent cell-mediated cytotoxicity (ADCC) assay (Fig. 3*D*). As mouse IgG1, S1V2-72 did not induce FcγRIV signaling in the same assay (*SI Appendix*, Fig. S5*B*), as expected (33). We conclude that Abs to the S1V2-72 epitope protect against influenza-driven pathology by Fcγ receptor–dependent mechanisms, rather than by neutralization.

### S1V2-72-Class Abs Share a Genetic Signature.

**Fig. 4.**
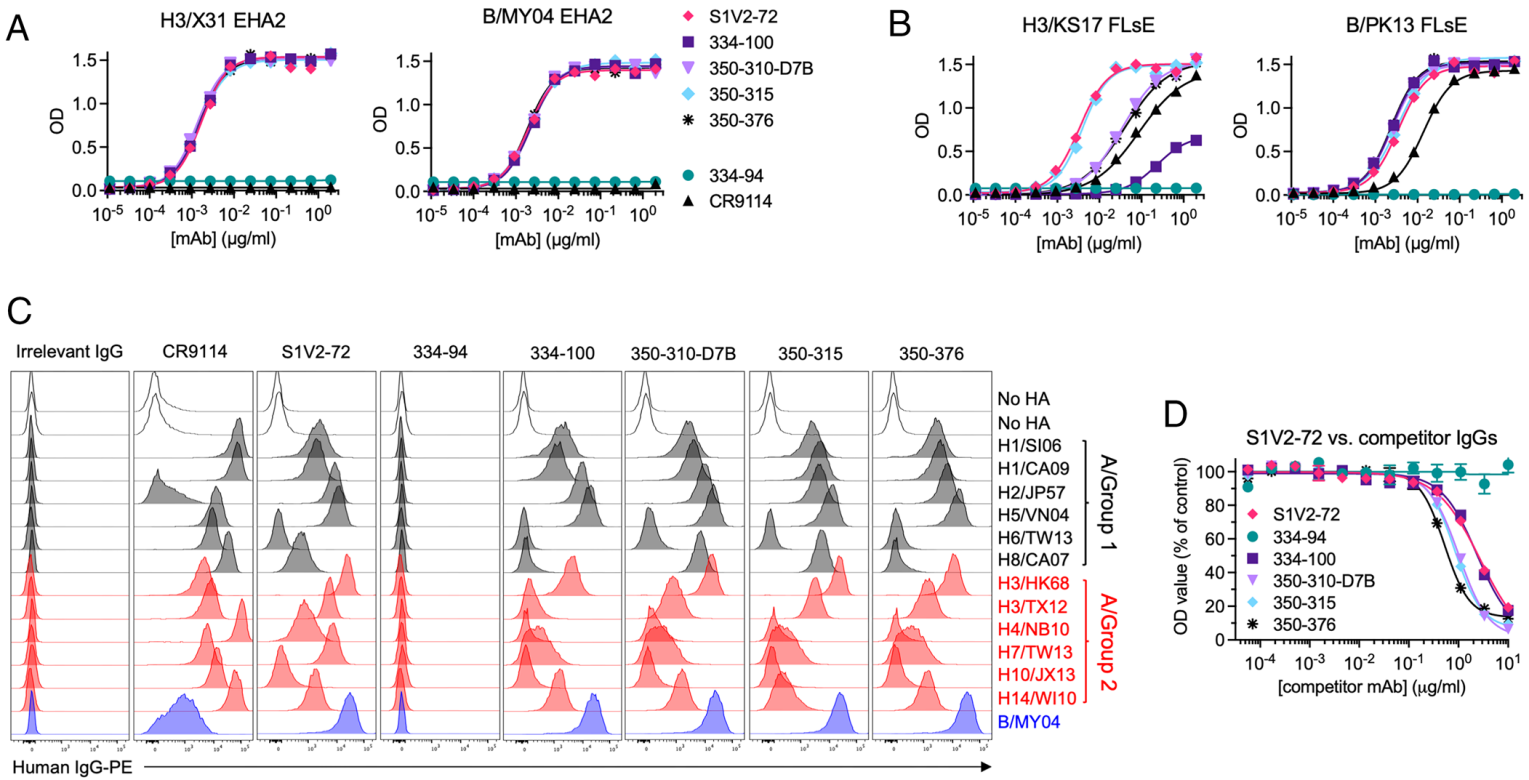
Characterization of mAbs identified by their S1V2-72-like genetic signature. (*A* and *B*) ELISA results showing rIgG binding to EHA2s (*A*) or HA0s (*B*). (*C*) Flow cytometry histograms depicting rIgG binding to K530 cell lines expressing recombinant, native HA on the cell surface. (*D*) Ab epitope overlap determined by competitive inhibition of binding in an ELISA. Each curve depicts the binding of S1V2-72 to B/MY04 EHA2-coated microplates in the presence of serially diluted competitor mAbs.

S1V2-72 and K06.18 are from different donors, but they compete for the same epitope, have similar breadth, and come from similar V(D)J rearrangements comprising an *IGHV1-2*-derived H chain with a 12-amino acid HCDR3, and an *IGLV2*-family L chain. To test whether such V(D)J rearrangements constitute a genetic signature for an S1V2-72-like public Ab response, we mined a dataset of 1,952 mAbs generated from putative HA-binding Bmem cells isolated from human donors 28 d after immunization with a chimeric HA vaccine (27). We selected 5 mAbs with H chains encoded by *IGHV1-2*, L chains encoded by *IGLV2*-family genes, and HCDR3s encompassing 11 to 14 amino acids. The five mAbs, named 334-94, 334-100, 350-310-D7B, 350-315, and 350-376, were from two donors (334 and 350), and included three singletons and a clonal dyad. Recombinant IgG versions of each mAb, except 334-94 (a singleton), bound H3/X31 and B/MY04 EHA2s (Fig. 4*A*), as well as B/PK13 and H3/KS17 HA0s (Fig. 4*B*) in ELISA. Moreover, except for 334-94, the mAbs bound B/MY04 plus many IAV group 1 and 2 rHAs expressed on the surface of K530 cell lines (Fig. 4*C*), although no mAb could match the full breadth of S1V2-72 for IAV HA subtypes (e.g., only S1V2-72 bound H4/NB10). Like S1V2-72, none of the new mAbs bound H6/TW13 or H10/JX13. Finally, the four new HA-reactive mAbs potently competed with S1V2-72 for EHA2 binding (Fig. 4*D*). Thus, a common genetic signature identifies many S1V2-72-like mAbs.

### Frequency of S1V2-72-Competing Bmem in Human Donors.

Whereas Bmem with breadth for group 1 and group 2 IAV plus IBV are rare (2/1,642 HA^+^ Nojima cultures; 0.1%), Bmem with epitopes that overlap the S1V2-72 epitope and hence compete, but bind with more restricted breadth, may be more frequent. To find Bmem of this kind, we screened Nojima culture supernatants containing HA-binding IgG (n = 860, from seven donors) for competition with S1V2-72. About 4% (33/860) of culture supernatant IgGs, including some from each donor, inhibited by ≥80% the binding of S1V2-72 to one or more HAs (*SI Appendix*, Fig. S6*A*). Six of the seven donors had Bmem (18/860) that inhibited S1V2-72 binding by ≥90%. The 18 most potent S1V2-72 competitors had substantial intrasubtypic breadth for historical H1, H3, or B HAs, and some even had heterosubtypic or cross-group breadth (*SI Appendix*, Fig. S6*B*). Sixteen of the 18 Bmem bound EHA2 forms of H3/X31 or B/MY04 (*SI Appendix*, Fig. S6*B*). The remaining two Bmem might also bind postfusion HA, but they are specific for group 1 HAs, which were not represented by any EHA2s in our screen. Nearly all (15/18) of the S1V2-72 competitors also reacted with LAH peptides from H5/VN04, H3/X31, and/or B/MY04 (*SI Appendix*, Fig. S6*C*), although S1V2-72 did not (Fig. 1*G*).

We selected four broadly binding S1V2-72-competing mAbs from different donors for further characterization as rIgGs. The rIgGs recapitulated the breadth of HA binding with the corresponding Nojima culture supernatant Abs (*SI Appendix*, Fig. S6*D*) and also substantially inhibited S1V2-72 binding to rHAs on Luminex beads (*SI Appendix*, Fig. S6*E*). Each S1V2-72 competitor had detectable heterosubtypic reactivity; the broadest, S5V6-P7F12, avidly bound HAs from most IAV group 1 subtypes and from both lineages of IBV, but interacted with IAV group 2 HAs weakly, if at all (*SI Appendix*, Fig. S6 *D* and *F*).

### The S1V2-72 Epitope Is Immunogenic in Mice.

**Fig. 5.**
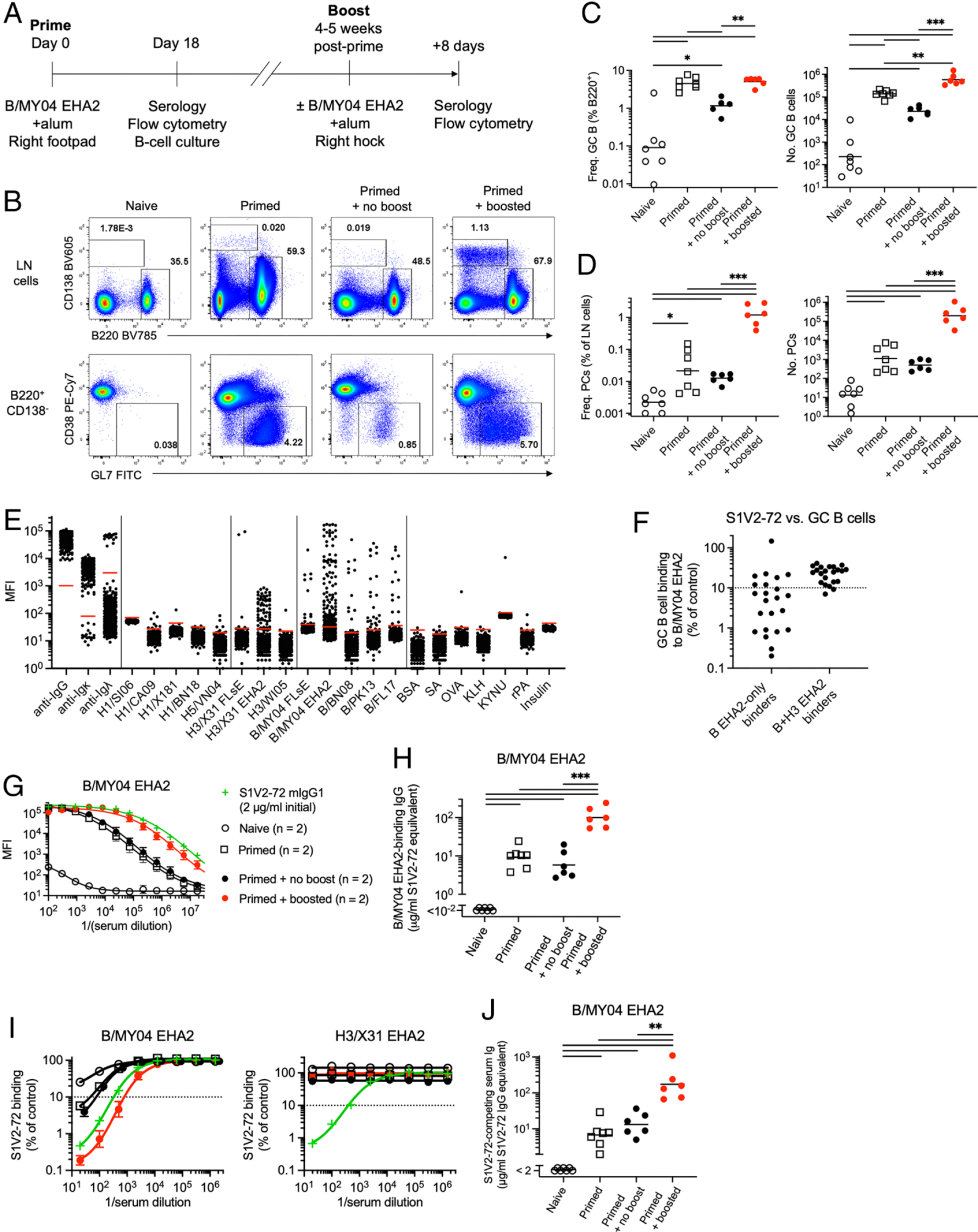
B/MY04 EHA2 vaccination elicits potent GC and serum Ab responses against the S1V2-72 epitope. (*A*) Schematic depicting the schedule for vaccinating mice and analyzing their immune response. (*B*) Representative flow cytometry dot plots depicting the frequencies of GC B cells and PCs in draining popliteal LNs from naive, primed (18 d postprime), primed and boosted (8 d postboost), or primed but unboosted (same timepoint as boosted) mice. (*C* and *D*) Aggregate frequencies and absolute numbers of GC B cells (*C*) and PCs (*D*) from mice treated as described in (*A*) and (*B*). Data were pooled from three independent experiments with two to three animals per group per experiment. (*E*) Luminex MFI values for culture supernatant IgG binding. Each symbol represents clonal IgG from a single GC B cell isolated 18 d postprime with B/MY04 EHA2. Data are from one experiment with three mice, representative of two independent experiments comprising five mice total. Horizontal red lines denote the binding threshold (mean plus 6 SD of signal from control wells containing no B cells). (*F*) Competitive inhibition by S1V2-72. S1V2-72 human IgG or irrelevant human IgG was preincubated with B/MY04 EHA2 Luminex beads, followed by addition of Nojima culture supernatant (mouse) IgGs. Competitive inhibition was calculated as mouse IgG binding in the presence of S1V2-72 as a percentage of mouse IgG binding in the presence of irrelevant human IgG. Values below the dotted horizontal line are >90% inhibited. We tested only clonal supernatant IgGs whose (uninhibited) binding MFI for B/MY04 EHA2 was ≥10^3^ (*E*). (*G*) Luminex MFI values for B/MY04 EHA2 binding by serially diluted serum IgG from mice treated as in (*A*). Each curve shows the geometric mean ± SEM. Binding by recombinant S1V2-72 mouse IgG1 standard (2 μg/mL initial, then serially threefold diluted) is shown for comparison. Data are from one experiment, representative of three independent experiments with similar results. (*H*) Concentrations of B/MY04 EHA2-reactive serum IgG, normalized to S1V2-72 mouse IgG1 standard Ab [as shown in (*G*)]. Data were pooled from three independent experiments with two to three mice per group per experiment. (*I*) Competitive inhibition by immune serum. Serially diluted serum was preincubated with B/MY04 EHA2 or H3/X31 EHA2 Luminex beads; then, S1V2-72 human IgG was added. Inhibition was calculated as the percentage of human IgG binding signal relative to control samples lacking mouse Ig. Each curve depicts the geometric mean ± SEM of the groups shown in (*G*). Values below the dotted horizontal line are >90% inhibited. Inhibition by recombinant S1V2-72 mouse IgG1 standard (2 μg/mL initial, then serially threefold diluted) is shown for comparison. (*J*) Total S1V2-72-competing B/MY04 EHA2-reactive serum Ig, normalized to S1V2-72 mouse IgG1 standard Ab [as shown in (*I*)]. Each symbol represents a single animal (*C*, *D*, *F*, *H*, and *J*). Solid black horizontal lines denote geometric means (*C*, *D*, *H*, and *J*). Asterisks (*C*, *D*, *H*, and *J*) denote statistically significant differences at *P* < 0.05 (*), *P* < 0.01 (**), and *P* < 0.001 (***). See *SI Appendix*, *Materials and Methods* for details of statistical analyses.

To determine whether vaccination with postfusion HA2 can elicit S1V2-72-like Abs, we immunized C57BL/6 J mice with B/MY04 EHA2 in alum adjuvant, then analyzed serum Ab and draining lymph node (LN) germinal center (GC) B cell and plasma cell (PC) responses 18 d later, or else 8 d after an ipsilateral boost with homologous antigen (Fig. 5*A*).

At 18 d postprime, draining LN GC B cell (B220^+^CD138^−^​CD38^lo^GL7^+^IgD^−^) numbers were ≥30-fold greater in B/MY04 EHA2-vaccinated mice than in naive controls, and remained ≥10-fold above background for at least 5 to 6 wk in primed animals (Fig. 5 *B* and *C*). LN PC (B220^−^CD138^+^) numbers were also modestly greater in most B/MY04 EHA2-primed animals than in naive controls, both at 18 d and 5 to 6 wk (Fig. 5 *B* and *D*). Eight days after boosting with homologous antigen, GC B cell and PC numbers were ~10-fold and ~100-fold greater, respectively, than in unboosted mice (Fig. 5 *B–D*).

To determine the antigen-specificity of GC B cells responding to primary immunization with B/MY04 EHA2, we sorted individual GC B cells 18 d postprime into single-cell Nojima cultures and subsequently analyzed the secreted clonal IgGs (34). In contrast to resting mature follicular B cells, of which ≤5% (7/151) bound HAs (and even then, only weakly), ~25% of primary GC B cells bound B/MY04 EHA2 (Fig. 5*E* and *SI Appendix*, Fig. S7*A* and Table S3). Of the B/MY04 EHA2-binders in the GC, ~25% also bound H3/X31 EHA2, while a separate subpopulation cross-reacted with IBV prefusion HAs (*SI Appendix*, Fig. S7*B* and Table S3). None of the GC B cells bound strongly to group 1 prefusion HA0s (Fig. 5*E*). S1V2-72 competed strongly with many IBV-restricted GC B cells for HA binding (Fig. 5*F*), indicating substantial epitope overlap, while most B + H3 cross-reactive cells were less sensitive to S1V2-72 competition (Fig. 5*F*), implying distinct epitopes. Thus, immunization with IBV EHA2 elicits GC B cell responses targeting multiple epitopes: some cells bind at least parts of the S1V2-72 epitope, but only for IBV; other GC B cells target one or more epitopes that do not overlap with that of S1V2-72, yet are present in the postfusion HA2 of H3/X31, B/MY04, and possibly other HAs not tested.

In addition to provoking substantial GC B cell responses, priming and boosting with B/MY04 EHA2 also elicited a high concentration of serum IgG that bound B/MY04 EHA2 and prefusion IBV HAs (Fig. 5 *G* and *H* and *SI Appendix*, Fig. S7 *C* and *D*). Vaccination also modestly but reproducibly raised the titer of H3/X31 EHA2-reactive serum IgG (*SI Appendix*, Fig. S7 *C* and *D*), which may have derived from the many GC B cells of low avidity for H3/X31 EHA2 (Fig. 5*E*). B/MY04 EHA2 immunization rarely yielded enhanced IgG titers against prefusion H3/X31, although a few animals developed detectable anti-H3/X31 FLsE responses (*SI Appendix*, Fig. S7 *C* and *D*). Serum IgG titers against prefusion group 1 HAs (H1/X181, H5/VN04) were, with a few exceptions, no different from those in naive controls (*SI Appendix*, Fig. S7 *C* and *D*). In most cases, the immune serum potently inhibited S1V2-72 binding to B/MY04 EHA2, but not H3/X31 EHA2 (Fig. 5 *I* and *J*).

## Discussion

Of previously studied HA Abs, CR9114 is the only one that protects broadly against both IAV and IBV disease. Isolation of CR9114 from a combinatorial display library raised the question of whether any native human Ab can bind diverse IAV and IBV HAs. We have now identified five clonal Abs, from four unrelated donors, with broad cross-reactivity for IAV and IBV HAs. These five Abs all appear to bind the same epitope, all derive from *IGHV1-2* and *IGLV2*-family genes, and all have short HCDR3s (11 to 13 amino acids). S1V2-72-like Abs thus collectively constitute a public Ab response. S1V2-72 protects against both IAV and IBV when constituted with an FcγR-activating constant region, supporting elicitation of S1V2-72-like Abs as a potential strategy for a more effective influenza vaccine. Moreover, that S1V2-72-like Abs recognize not only HAs from IAV and IBV strains currently circulating in human populations but also HAs from subtypes that currently circulate solely among animals, indicates that a vaccine provoking an S1V2-72-like humoral response may impart prepandemic immunity as well.

S1V2-72 might be able to direct immune clearance of virions and infected cells by recognizing its epitope on HA in either the pre- or postfusion conformation. We showed that S1V2-72 can bind its epitope as present on solubilized prefusion ectodomains, indicating that conformational fluctuations may transiently expose the otherwise occluded epitope, analogous to the molecular “breathing” that allows HA head-interface Abs access to their epitopes (10). At present, we cannot say whether such conformational flexibility also explains how S1V2-72-like Abs recognize membrane-anchored HA, where epitope access may also be hindered by proximity to the cell membrane.

The presence of postfusion HA on the surface of infected cells might also account for at least part of the protective efficacy of postfusion-HA directed Abs. The recently identified mAb, LAH31 (21), binds a conformational epitope unique to postfusion HA, yet LAH31 avidly binds cell-surface HA on IAV-infected cell lines in vitro, and protects mice against IAV disease, implying that there is postfusion HA on infected cells in vivo. Because of its avidity for postfusion HA2, S1V2-72 could also confer in vivo protection by recognizing postfusion HA directly. Like S1V2-72 and LAH31, several HA mAbs with cryptic epitopes exposed at pH < 5.5 also protect against influenza-associated weight loss by FcγR-dependent mechanisms (19), providing further evidence that humoral responses to postfusion HA epitopes might be important contributors to immune control of IAV and IBV infections.

How can postfusion HA appear on the cell surface? HA transitions to the postfusion form in acidifying (pH < 5.5) endosomes (35), and it is presumably degraded later when the endosome merges with a lysosome. Takahashi and colleagues have proposed (18, 21) three non-mutually exclusive hypotheses for how extracellular postfusion HA might arise: release from lysed infected cells, conformational transition during inflammation-driven extracellular acidification, and conformational transition during endosomal recycling in follicular dendritic cells. All three hypotheses explain how the immune system could be sensitized to postfusion HA, and the second mechanism could account for how postfusion HA Abs such as LAH31 could control an influenza infection, if the postfusion HA decorated the surface of infected cells. However, extracellular acidification cannot explain why LAH31 brightly labels cells infected with IAV in vitro (21), where the extracellular pH is buffered >6. Instead, we suggest that postfusion HA appears regularly on the surface of infected cells after escaping from acidified early endosomes via endocytic recycling pathways (36). In this way, expression of postfusion HA on cell membranes would be independent of extracellular pH and would identify infected cells for immune lysis.

Whatever the mechanism of immune sensitization, human humoral responses to postfusion HA are more frequent than previously appreciated. We and others (18, 21) have now shown that many human donors harbor within their Bmem cell populations Abs that bind postfusion HA, some of which have epitopes overlapping that of S1V2-72. Postfusion epitopes must be readily immunogenic because mice vaccinated with postfusion HA2 generate robust GC and serum Ab responses against the S1V2-72 epitope and at least one other nonoverlapping postfusion epitope. Both S1V2-72-competing and noncompeting EHA2-reactive cells constitute substantial fractions of the resultant GC B cell population and can bind both IAV and IBV, suggesting that EHA2 vaccination might be a component of a strategy for achieving broad protection against influenza.

We cannot determine whether the human Bmem cells that bound postfusion structures in our study were elicited by infection or by vaccination. The immune system is efficiently sensitized to postfusion HA epitopes during influenza infection (18, 19), but the licensed inactivated influenza vaccines administered to donors S1 and KEL06 are prepared under conditions predicted largely to preserve HA in its prefusion conformation. This expectation is supported by the observation that whereas mice immunized with acid-treated HA-split vaccine (i.e., postfusion HA) mount a robust humoral response to conserved epitopes preferentially exposed in the postfusion state, vaccination with nonacidified split vaccine elicits no such response (18). Nevertheless, S1V2-72 binds solubilized prefusion HA0, and LAH31 also modestly binds commercial vaccine HAs (21), so we cannot rule out that seasonal vaccination might have contributed to the development of the postfusion HA Abs we and others have identified. Human V_H_1-2 and V_λ_2-23 knockin mice (37, 38) may be useful for testing vaccination regimens aiming to recapitulate the development of S1V2-72-like Abs more efficiently than current seasonal vaccines.

The sequence of antigen exposures that elicited S1V2-72 or K06.18 cannot be deduced because the inferred germline versions of S1V2-72 and K06.18 bind multiple IAV and IBV HAs. The breadth of the inferred germline Abs, paired with the contributions of HCDR1 and HCDR2 to the paratope, implies that the capacity of S1V2-72-like Abs to bind both IAV and IBV HAs may be germline-encoded. In contrast, the CR9114 germline precursor binds H1s, but must acquire five specific amino acid substitutions to bind H3s, and nine substitutions to bind B HAs (39). Future efforts will focus on identifying multimember lineages of S1V2-72-like Abs, to more accurately determine germline CDR3 sequences and the antigen specificity of the germline Abs.

Research on vaccines for influenza and many other viruses concentrates largely on eliciting neutralizing Abs, which prevent infection and often have greater protective potency than non-neutralizing Abs. But a central conundrum of neutralizing Abs is their tendency to select for viral mutations that enable escape from immune control. Because the number of neutralizing epitopes is generally a modest fraction of the total number of potential epitopes on viral proteins, relatively few escape mutations are needed to evade neutralizing humoral responses (40, 41). Non-neutralizing, HA-directed Abs with potent Fc effector activities protect mice from morbidity and mortality after receiving an otherwise lethal dose of influenza virus (10, 42). Therefore, to confer immunity that resists antigenic drift, an influenza vaccine might need to broaden its epitopic coverage beyond neutralization and include additional tiers of control, including eliciting Abs to conserved, non-neutralizing epitopes.

## Materials and Methods

The full protocols for studies involving human subjects were approved by the Duke University Institutional Review Board or Boston University Institutional Review Board. PBMCs were obtained from human donors KEL01 (male, age 39), KEL03 (female, age 39), KEL06 (female, age 35), S1 (female, age 51 to 55), S5 (male, age 21 to 25), S8 (female, age 26 to 30), S9 (female, age 51 to 55), and S12. Written informed consent was obtained from all subjects.

Soluble rHAs were expressed in baculovirus-infected insect cells or *Escherichia coli* cells. LAH-Fc fusion proteins and rIgGs were expressed in Expi293 cells. IgG^+^ Bmem cells that bound fluorescently tagged rHA were isolated from healthy human donors before and 1 to 2 wk after immunization with seasonal influenza vaccine. IgG-containing supernatants from cultures of individual Bmem cells were screened for reactivity to H1, H5, H3, and B HAs in Luminex assays, and V(D)J rearrangements encoding clonal IgGs of interest were recovered from cultured B cells by RT-PCR. Broadly binding clonal IgGs were expressed as rIgGs for further characterization of binding breadth by ELISA, Luminex assay, flow cytometry, and BLI assays. The protective activity of S1V2-72 was tested in vitro by standard microneutralization assays with H1N1, H3N2, and IBV, and by an ADCC assay comprising coincubated S1V2-72 or control mAbs (as mouse rIgG2c) plus HA-expressing target cells and effector cells in which luciferase activity was stimulated by mouse FcγRIV activation. In vivo protection was determined by i.p. injecting mouse IgG2c or IgG1 versions of S1V2-72 or control mAbs into female C57BL6/J mice 3 h prior to lethal intranasal challenge with H3N2 or IBV. Animals were weighed and monitored for survival daily. The immunogenicity of the S1V2-72 epitope was determined by footpad immunization of female C57BL6/J mice with B/MY04 EHA2 plus alum. Some mice were boosted with homologous antigen in the hock 4 to 5 wk after priming. Eighteen days postprime or 8 d postboost, immune responses in the draining LN were analyzed by flow cytometry and single B cell culture. Immune sera were also collected for analysis by ELISA and Luminex. The specificity of mouse GC B cells and serum IgG for the S1V2-72 epitope was determined by Luminex competitive binding assay. A competitive binding assay was also used to screen supernatants from cultured human Bmem cells, to identify S1V2-72-competing IgGs. For cryo-EM structural analysis, S1V2-72 Fab was incubated with B/MY04 EHA2 trimer in a 9:1 ratio to achieve 3 Fabs per trimer, and the complex was purified by gel filtration. S1V2-72:EHA2 complex was deposited onto 300 mesh Quantifoil Au 0.6/1.0 grids, blotted, and vitrified in liquid ethane. Micrographs were recorded on a 300 kV Titan Krios G3i microscope with a K3 direct electron detector. Dose-fractionated images were gain normalized, aligned, dose-weighted, and summed using MotionCor2. CryoSPARC was used for contrast transfer function and defocus value estimation, particle picking, 2D classification, 3D reconstruction, and refinement. Atomic models of B/MY04 EHA2 and S1V2-72 Fab were predicted with AF2 and fit to the cryoEM map with UCSF ChimeraX and Phenix. Complete details of methods are described in *SI Appendix*, *Materials and Methods*.

## Supplementary Material

Appendix 01 (PDF)

## Data Availability

V(D)J sequences for S1V2-72 and K06.18 are available at GenBank (accession numbers OR542561–OR542564) (43–46). The map of the cryo-EM reconstruction of S1V2-72 Fab bound to EHA2 is available at the Electron Microscopy Data Bank (accession number EMD-42149) (47). Coordinates for the docked model of S1V2-72 bound to EHA2 is available at the Protein Data Bank (accession number 8UDG) (48).
