# Supplementary material for: Protective human antibodies against a conserved epitope in pre- and postfusion influenza hemagglutinin
Source: Proc Natl Acad Sci U S A. 2023 Dec 26;121(1):e2316964120. doi: 10.1073/pnas.2316964120 (PMC10769852; doi:10.1073/pnas.2316964120)
Supplement: Supplementary file 1 — Appendix 01 (PDF) [file pnas.2316964120.sapp.pdf]

PNAS

Supporting Information for

Protective human antibodies against a conserved epitope in pre- and postfusion influenza hemagglutinin

Joel Finney, Annie Park Moseman, Susan Kong, Akiko Watanabe, Shengli Song, Richard M. Walsh Jr., Masayuki Kuraoka, Ryutaro Kotaki, E. Ashley Moseman, Kevin R. McCarthy, Dongmei Liao, Xiaoe Liang, Xiaoyan Nie, Olivia Lavidor, Richard Abbott, Stephen C. Harrison, Garnett Kelsoe

Correspondence:
Stephen C. Harrison
Email: harrison@crystal.harvard.edu

Garnett Kelsoe
Email: ghkelsoe@duke.edu

This PDF file includes:

Supporting Materials and Methods
Figures S1 to S7
Tables S1 to S3
SI References

SUPPORTING MATERIALS AND METHODS

Human subjects

KEL01 and KEL03 received the trivalent inactivated seasonal influenza vaccine (TIV) 2014-2015 Fluvirin, which contained A/Christchurch/16/2010, NIB-74 (H1N1), A/Texas/50/2012, NYMC X-223 (H3N2), and B/Massachusetts/2/2012, NYMC BX-51B. KEL06 received the TIV 2015-2016 Flucelvax, which contained A/Brisbane/10/2010 (H1N1), A/South Australia/55/2014 (H3N2), and B/Utah/9/2014. Blood was drawn on day 14 post-vaccination, and PBMCs isolated by centrifugation over Ficoll density gradients (SepMate-50 tubes, StemCell Tech) were frozen and kept in liquid nitrogen until use.

Subjects S1, S5, S8, S9, and S12 met all of the following inclusion criteria for a study approved by the Boston University Institutional Review Board: between 18 and 65 years of age; in good health, as determined by vital signs [heart rate (<100 bpm), blood pressure (systolic ≤ 140 mm Hg and ≥ 90 mm Hg, diastolic ≤ 90 mm Hg), oral temperature (<100.0 °F)] and medical history to ensure existing medical diagnoses/conditions are not clinically significant; can understand and comply with study procedures, and; provided written informed consent prior to initiation of the study. Exclusion criteria included: 1) life-threating allergies, including an allergy to eggs; 2) have ever had a severe reaction after influenza vaccination; 3) a history of Guillain-Barre Syndrome; 4) a history of receiving immunoglobulin or other blood product within the 3 months prior to vaccination in this study; 5) received an experimental agent (vaccine, drug, biologic, device, blood product, or medication) within 1 month prior to vaccination in this study or expect to receive an experimental agent during this study; 6) have received any live licensed vaccines within 4 weeks or inactivated licensed vaccines within 2 weeks prior to the vaccination in this study or plan receipt of such vaccines within 2 weeks following the vaccination; 7) have an acute or chronic medical condition that might render vaccination unsafe, or interfere with the evaluation of humoral responses (includes, but is not limited to, known cardiac disease, chronic liver disease, significant renal disease, unstable or progressive neurological disorders, diabetes mellitus, autoimmune disorders and transplant recipients); 8) have an acute illness, including an oral temperature greater than 99.9°F, within 1 week of vaccination; 9) active HIV, hepatitis B, or hepatitis C infection; 10) a history of alcohol or drug abuse in the last 5 years; 11) a history of a coagulation disorder or receiving medications that affect coagulation. Subjects S1, S5, S8, S9, and S12 received seasonal influenza vaccination during three consecutive North American flu seasons (2015-2016, 2016-2017, 2017-2018), and had blood drawn on day 0 (pre-vaccination) and day 7 (post-vaccination) each year. During the 2015-2016 season, the subjects received the TIV Fluvirin, which contained A/reassortant/NYMC X-181 (California/07/2009 x NYMC X-157) (H1N1), A/South Australia/55/2014 IVR-175 (H3N2), and B/Phuket/3073/2013. During the 2016-2017 season, the subjects received the quadrivalent inactivated vaccine Flucelvax, containing A/Brisbane/10/2010 (H1N1), A/Hong Kong /4801/2014 (H3N2), B/Utah/9/2014, and B/Hong Kong/259/2010. During the 2017-2018 season, the subjects received the quadrivalent inactivated vaccine Flucelvax, containing A/Singapore/GP1908/2015 IVR-180 (H1N1), A/Singapore/GP2050/2015 (H3N2), B/Utah/9/2014, and B/Hong Kong/259/2010.

Mice

Female C57BL/6J mice (#000664) were obtained from the Jackson Laboratory and maintained under specific pathogen-free conditions at the Duke University Animal Care Facility. Eight- to 12-week-old mice were immunized in the hind footpad (prime) or ipsilateral hock (boost) with 10 µg of B/MY04 EHA2 adjuvanted with Alhydrogel® (Invivogen). All experiments involving animals were approved by the Duke University Institutional Animal Care and Use Committee.

Cell line culture

Unless otherwise noted, mammalian cell lines were maintained in static cultures at 37°C with 5% CO_2 in a humidified incubator, and culture reagents were from Gibco. MS40L-low feeder cells (*Mus musculus*)(1, 2) were expanded from frozen aliquots in Iscove's Modified Dulbecco's Medium containing 10% HyClone FBS (Cytiva), 2-mercaptoethanol (55 μM), penicillin (100 units/ml), and streptomycin (100 μg/ml). NB21.2D9 cells (*Mus musculus*)(3) were cultured in Dulbecco's Modified Eagle Medium (DMEM) plus 10% FBS, penicillin, streptomycin, and 1× MEM non-essential amino acids. Expi293F cells (*Homo sapiens*; Thermo Fisher) were cultured in Expi293 Expression Medium plus penicillin and streptomycin, at 8% CO_2 with shaking. K530-derived cell lines (*Homo sapiens*) (4) were cultured in B-cell medium (BCM: RPMI-1640 medium plus 10% FBS, 2-mercaptoethanol (55 μM), penicillin, streptomycin, HEPES (10 mM), sodium pyruvate (1 mM), and MEM nonessential amino acids). Madin-Darby canine kidney (MDCK) cells (*Canis lupus familiaris*) were maintained in DMEM plus 10% FBS. High Five cells (BTI-TN-5B1-4; *Trichoplusia ni*; Thermo Fisher) were maintained in ESF 921 medium (Expression Systems) at 28°C in spinner flasks in air. Cell lines were not subject to authentication.

Recombinant HA expression and purification

Except where explicitly noted otherwise in the figures or text, all recombinant HAs were full-length soluble ectodomain (FLSE) trimers. Recombinant HAs (except for EHA2s) were expressed by infection of insect cells with recombinant baculovirus as described (5-8). In brief, synthetic DNA corresponding to the full-length ectodomain or globular HA-head were subcloned into a pFastBac vector modified to encode a C-terminal thrombin cleavage site, a T4 fibritin (foldon) trimerization tag, and a hexa-His tag. The resulting baculoviruses produce HA trimers and trimeric HA heads. Supernatant from recombinant baculovirus-infected High Five cells was harvested 72 h post-infection and clarified by centrifugation. Proteins were purified by adsorption to cobalt-nitrilotriacetic acid (Co-NTA) agarose resin (Takara), followed by a wash in buffer A (10 mM Tris, 150 mM NaCl, pH 7.5) plus 5 mM imidazole, elution in buffer A plus 350 mM imidazole (pH 8), and gel filtration chromatography on a Superdex 200 column (GE Healthcare) in buffer A.

The following HAs were thus produced: H1N1 A/USSR/90/1977 (H1/USSR77), H1N1 A/Massachusetts/1/1990 (H1/MA90), H1N1 A/Florida/2/1993 (H1/FL93), H1N1 A/Solomon Islands/03/2006 (H1/SI06), H1N1 A/California/04/2009 (H1/CA09), H1N1 A/reassortant/NYMC X-181 (California/07/2009 × NYMC X-157) (H1/X181), H1N1 A/Michigan/45/2015 X-275 (H1/MI15 X-275), H1N1 A/Brisbane/2/2018 IVR-190 (H1/BN18 IVR-190), H3N2 A/Aichi/2/1968 (H3/X31), H3N2 A/Texas/1/1977 (H3/TX77), H3N2 A/Bangkok/01/1979 (H3/BK79), H3N2 A/Philippines/2/1982 (H3/PH82), H3N2 A/Leningrad/360/1986 (H3/LG86), H3N2 A/Wisconsin/67/2005 (H3/WI05), H3N2 A/South Australia/55/2014 IVR-175 (H3/IVR175), H3N2 A/Hong Kong/4801/2014 (H3/HK14), H3N2 A/Singapore/INFIMH-16-0019/2016 (H3/SP16), H3N2 A/Kansas/14/2017 X-327 (H3/KS17) and point mutants thereof (9), H4 A/American black duck/New Brunswick/00464/2010 (H4/NB10), H5 A/Vietnam/1203/2004 (H5/VN04), B/Malaysia/2506/2004 (B/MY04), B/Brisbane/60/2008 (B/BN08), B/Phuket/3073/2013 (B/PK13), B/Florida/84/2017 (B/FL17); and trimeric, head-only rHA constructs of H3/WI05 (H3/WI05-h), H3 A/Johannesburg/33/1994 (H3/J'burg-h) and H3/X31 (H3/X31-h).

EHA2 expression and purification

Synthetic DNA encoding recombinant post-fusion HA2 (10) from B/MY04 (B/MY04 EHA2) or H3/X31 (H3/X31 EHA2) was cloned into a pET vector modified to include an N-terminal hexa-His tag and tobacco etch virus (TEV) protease cleavage site. Plasmid-transformed Rosetta(DE3) *E. coli* clones (Novagen) were inoculated into 50 ml cultures of 2×YT or LB medium and grown overnight at 37°C with shaking. The following morning, the 50 ml cultures were used to inoculate 1L cultures of 2×YT or LB medium. The 1L cultures were incubated at 37°C with shaking until the OD600 reached 0.6-0.8, at which point EHA2 expression was induced by addition of isopropyl β-D-1-thiogalactopyranoside (1 mM final). The cultures were then incubated overnight at 18°C with shaking. Cells were harvested by centrifugation, suspended in lysis buffer (40 mM Tris pH 8, 500 mM NaCl, 2 mM 2-mercaptoethanol, 10 mM imidazole), and disrupted by ultrasonication on ice. The EHA2-containing lysate was clarified by centrifugation at 40,000×*g* for 30 min at 4°C, then EHA2 was purified from the supernatant fluid by adsorption to Co-NTA agarose resin, followed by extensive washing in 40 mM Tris pH 8 plus 100 mM NaCl, 10% glycerol, 2 mM 2-mercaptoethanol, and 30 mM imidazole. EHA2 was eluted in buffer A plus 400 mM imidazole. For immunization and cryo-EM studies, the hexa-His tag was then removed by incubating the EHA2 with TEV protease, and the tag-less EHA2 was isolated by gel filtration chromatography on a Superdex 200 column in PBS.

Generation of HA-expressing K530 cell lines

Monoclonal, fluorescent protein-barcoded K530 cell lines stably expressing HAs from A/California/04/2009 (H1/CA09; GenBank accession FJ966082), B/Malaysia/2506/2004 (B/MY04; GenBank CY038287), or B/reassortant/NYMC BX-51B(Massachusetts/2/2012 x NYMC BX-46) (B/MA12; GenBank KF752446) were prepared by lentiviral transduction and single-cell sorting, as described (4).

Flow cytometry

Human Bmem cells were isolated by flow cytometry as described (11). PBMCs in DMEM containing 10% FBS were blocked with mouse IgG1 (MG1K; Rockland) and then labeled with anti-human IgM-FITC (MHM-88), anti-CD3-PE-Cy5 (UCHT1), anti-CD14-Tri (TuK4), anti-CD16-PE-Cy5 (3G8), anti-CD19-PE-Cy7 (HIB19), anti-IgG-APC (G18-145), anti-IgD-APC-Cy7 (IA6-2), anti-CD27-BV421 (M-T271), and anti-CD24-BV510 (ML5), purchased from BD Biosciences, BioLegend, or Thermo Scientific. To isolate HA-binding Bmem cells from donors KEL01, KEL03, and KEL06, PE-labeled H3/WI05 was prepared using R-Phycoerythrin Labeling Kit-NH2 (Dojindo). To isolate HA-binding Bmem cells from donors S1, S5, S8, S9, S12, and in some experiments KEL01 and KEL03, we used a mixture of PE-labeled H1/X181, PE-labeled H3/IVR175, and PE-labeled B/PK13. HA-binding (PE^{+}) $CD19^{+}CD27^{+}CD24^{hi}IgD^{-}IgM^{-}IgG^{+}$ cells were sorted on a FACS Vantage cytometer with Diva software (BD Biosciences). Flow cytometric data were analyzed with FlowJo software (Treestar Inc.). Doublets were excluded by FSC-A vs. FSC-H gating. Cells positive for 7-AAD (BD Bioscience) or for CD3, CD14, or CD16 expression were also excluded.

Mouse popliteal LNs were dispersed by gentle grinding between frosted glass slides. Dispersed cells were suspended in DMEM plus 10% FBS, 2-mercaptoethanol (55 μM), 2 mM additional L-glutamine, penicillin, and streptomycin, then labeled with fluorophore-conjugated Abs: anti-B220 BV785 (RA3-6B2, BioLegend), anti-CD38 PE-Cy7 (90, BioLegend), GL7 FITC (BD Biosciences), anti-CD138 BV605 (281-2, BD), and anti-IgD BV480 (11-26c.2a, BD). GC B

cells were identified as B220^{hi}CD138^{-}CD38^{lo}GL-7^{+}IgD^{-}, mature follicular B cells as B220^{hi}CD138^{-}CD38^{hi}GL-7^{-}IgD^{+}, and PCs as B220^{-}CD138^{+}. Dead cells and doublets were excluded from analysis based on propidium iodide (Sigma-Aldrich) staining and FSC-A/FSC-H gating, respectively. Labeled cells were analyzed or sorted into culture plates with a BD FACSymphony A5 analyzer or BD FACSymphony S6 sorter.

K530-derived cell lines were thawed from cryopreserved aliquots and expanded in culture for ≥3 days. Pooled K530 cells were incubated at room temperature (RT) for 25-30 min with 2 μg/ml anti-HA rIgGs or irrelevant human IgG (151L, Southern Biotech) diluted in PBS plus 2% fetal bovine serum. After washing, cells were labeled with 2 μg/ml PE-conjugated goat anti-human IgG (Southern Biotech) for 20-30 min at RT. Cells were then washed and analyzed with a BD FACS Canto II, a BD FACSymphony A5, or a BD LSRII flow cytometer.

Human single B-cell culture (Nojima culture)

Human Bmem cells were expanded in the presence of MS40L-low feeder cells as described (1, 2, 11). Single human Bmem cells were directly sorted into separate wells of 96-well plates and cultured with MS40L-low feeder cells in BCM supplemented with exogenous recombinant human IL-2 (50 ng/ml), IL-4 (10 ng/ml), IL-21 (10 ng/ml) and BAFF (10 ng/ml; all Peprotech). Cultures were maintained at 37°C with 5% CO_2. Half of the culture medium was replaced twice weekly with fresh BCM plus cytokines. On culture day 25, culture supernatants were harvested for screening the reactivity of secreted clonal IgGs. Expanded clonal B cells were frozen for subsequent V(D)J sequence analysis.

Mouse single B-cell culture (Nojima culture)

Mouse GC B cells were expanded in the presence of NB21.2D9 feeder cells essentially as described (3). Single B cells were directly sorted into separate wells of 96-well plates pre-seeded with NB21.2D9 cells in 200 μl BCM supplemented with recombinant murine IL-4 (2 ng/ml; Peprotech). Cultures were maintained at 37°C with 5% CO_2. After two days of culture, 100 μl of culture medium was removed and replaced with 200 μl of fresh BCM (without IL-4). On culture days 3, 5, 6, 7 and 8, 200 μl of culture medium was replaced with fresh BCM. On culture day 10, culture supernatants were harvested for screening the reactivity of secreted clonal IgGs.

Ab *V(D)J* rearrangement amplification and analysis

Rearranged *V(D)J* gene sequences for human Bmem cells from single-cell cultures were obtained by RT-PCR as described (11, 12). DNA sequencing was performed at the Duke University DNA Analysis Facility. V(D)J rearrangements were identified with Cloanalyst (13) and IMGT/V-QUEST (14).

Recombinant IgG expression and purification

DNA encoding H- or L-chain variable domains was cloned into expression vectors harboring the constant regions of mouse or human IgG1, Igκ, Igλ, or mouse IgG2c. IgGs were produced by transient transfection of Expi293F cells with the Expifectamine 293 transfection kit (Thermo Fisher), according to the manufacturer's instructions. Five days post-transfection, supernatants were harvested, clarified by low-speed centrifugation, mixed 1:1 with Protein G binding buffer (for mouse IgG1) or Protein A binding buffer (for human IgG1 or mouse IgG2c), and incubated overnight with Pierce Protein G or Protein A agarose resin (Thermo Fisher). The resin was collected in a chromatography column, washed with binding buffer, eluted in Pierce

IgG Elution Buffer (Thermo Fisher), neutralized by 1M Tris (pH 9), and dialyzed into PBS. IgG concentrations were determined with a NanoDrop spectrophotometer (Thermo Fisher).

Recombinant Fab purification

S1V2-72 Fab was generated from recombinant S1V2-72 human IgG1 with the Pierce Fab Preparation Kit (Thermo Fisher), according to the manufacturer's instructions.

LAH fusion proteins

LAH peptides from H3/X31 (amino acid sequence: RIQDLEKYVEDTKIDLWSYN AELLVALENQHTIDLTDSEMNKLFEKTRRQLRENA), H5/VN04 (ERRIENLNKKMEDGFL DVWTYNAELLVLMENERTLDFHDSNVKNLYDKVRLQLRDNA), or B/MY04 (HNEILELDEKVDDLRADTISSQIELAVLLSNEGIINSEDEHLLALERKLKKMLGPS) were expressed as human IgG1 Fc fusion proteins in Expi293F cells and purified with Protein A resin (Thermo Fisher) according to the manufacturer's protocol. Purified LAH-Fc fusion proteins were stored in PBS at 4°C.

Multiplex bead assay

The specificity and avidity of rIgGs and clonal IgGs in culture supernatants were determined by Luminex multiplex assay (Luminex Corp.)(11, 15). Culture supernatants or rIgGs were diluted in Luminex assay buffer (PBS plus 1% BSA, 0.05% NaN_3 and 0.05% Tween20) plus 1% non-fat milk and incubated for 2 h at RT (or overnight at 4°C) with a mixture of antigen-coupled microsphere beads in 96-well filter-bottom plates (Millipore). After washing with assay buffer, beads were incubated at RT for 1 h (or overnight at 4°C) with PE-conjugated rat anti-mouse IgG1 (SB77e), mouse anti-human IgG Fc (JDC-10), goat anti-human IgG, goat anti-mouse IgG (all from Southern Biotech), or rat anti-mouse IgG2a (RMG2a-62, BioLegend). After washing, the beads were suspended in assay buffer and analyzed on a Bio-Plex 3D Suspension Array System (Bio-Rad). The following antigens were coupled with carboxylated beads (Luminex Corp): BSA, goat anti-human Igκ, goat anti-human Igλ (both Southern Biotech), goat anti-human IgG Fc (Jackson ImmunoResearch), and rHAs.

ELISA

High-binding 96- or 384-well microplates were coated with 2 μg/ml protein in 0.1 M sodium carbonate buffer (pH 9) overnight at 4°C. For quantitation of secreted IgG in B-cell culture supernatants, the coating proteins were goat anti-human Igκ plus goat anti-human Igλ, or goat anti-mouse Igκ plus goat anti-mouse Igλ (all Southern Biotech). Otherwise, the coating proteins were rHA constructs. After washing with PBS plus 0.1% Tween20, the plates were blocked with PBS plus 0.5% BSA at RT for ≥30 min, then culture supernatants or rIgGs diluted in PBS plus 0.5% BSA and 0.1% Tween20 were added to the plates and incubated for 2 h at RT, or overnight at 4°C. After extensive washing, bound Abs were detected with goat anti-human IgG-HRP, goat anti-mouse IgG-HRP, or a mixture of goat anti-human Igκ-HRP and goat anti-human Igλ-HRP (all Southern Biotech) diluted in PBS plus 0.5% BSA and 0.1% Tween20. After washing, HRP activity was detected with the TMB substrate kit (BioLegend) and a Spectramax plate reader (Molecular Devices). Background signal at 650 nm was subtracted from the signal at 450 nm to calculate the OD450.

Competitive inhibition assay

Ab epitope mapping was performed by competitive binding inhibition in ELISA or Luminex assay (11, 15). In all experiments, competitor IgGs were from a different species than the analyte Ab (mouse vs. human, or vice versa). Generally, serially diluted competitor rIgGs or mouse sera were incubated with HA-coated substrate (either Luminex microspheres or ELISA plates) for 2 h at RT or overnight at 4°C. Analyte Ab (either K06.18 rIgG or S1V2-72 rIgG) was then added at a fixed concentration to each well, and incubated with the competitor Abs and HA substrate for 2 h at RT. After washing, bound analyte Ab was detected either by mouse anti-human IgG Fc-PE (JDC-10, SouthernBiotech), rat anti-mouse IgG2a-PE (RMG2a-62, BioLegend) or goat anti-mouse IgG Fc-HRP (SouthernBiotech).

In some experiments, the competitor Igs were diluted (1:10) supernatants from cultures of human Bmem cells, rather than rIgGs. Culture supernatants containing HA-nonbinding IgG were used as non-inhibiting controls.

To map the epitopes of mouse GC B cells elicited by immunization with B/MY04 EHA2, S1V2-72 human rIgG or irrelevant human IgG (151L, SouthernBiotech) was incubated with B/MY04 EHA2-conjugated microspheres for ≥2 h, then diluted (1:10 final) supernatants from cultures of mouse GC B cells were added to each well and incubated for 2 h at RT. The final concentration of S1V2-72 or irrelevant IgG was 10 μg/ml. After washing, bound mouse Ab was detected by rat anti-mouse IgG1-PE (SB77e, SouthernBiotech). To calculate binding as a percent of control, GC B-cell IgG binding signal in the presence of S1V2-72 was divided by GC B-cell IgG binding signal in the presence of irrelevant IgG, then multiplied by 100.

Biolayer interferometry (BLI)

BLI experiments were performed on a BLItz label-free protein analysis system (ForteBIO). All measurements were in Luminex assay buffer at RT. Purified B/MY04 FLSE or EHA2 was immobilized on Ni-NTA biosensors (Sartorius), and Fabs or IgGs were titrated to determine relative association rates. Alternatively, IgGs were immobilized on Protein A biosensors (Sartorius), and B/MY04 FLSE was titrated to determine relative association rates.

Mouse ADCC reporter assay

The potential for mAbs to mediate ADCC activity was determined using the Mouse FcγRIV ADCC Bioassay (Promega) according to manufacturer's instructions. Briefly, cloned H3/X31- or B/MY04-expressing K530 cells (target cells) were dispensed at 2.5×10^4 cells/well into white, flat-bottom 96-well assay plates (Corning #3917). Serially diluted mouse IgG2c Abs were added to the target cells and incubated at RT for 15 min. Effector cells expressing mouse FcγRIV were added to the wells for an effector:target ratio of 3:1, then the plate was incubated at 37°C, 5% CO_2 for 6 h. Finally, Bio-Glo Reagent was added to the plate and incubated for 15 min at RT. Luminescence was detected with a Synergy HTX multimode plate reader (BioTek). Background signal from wells containing no cells was subtracted from the data, and fold-induction was calculated as the quotient of signal in wells containing Ab divided by the signal in wells containing no Ab. Antibodies were assayed in duplicate.

Virus propagation

Influenza viruses A/Solomon Islands/3/2006 (H1N1), A/Aichi/2/68, X-31 (H3N2), and B/Malaysia/2506/2004 were propagated in embryonated, specific-pathogen-free, chicken eggs (Sunrise Farms Inc., Catskill, NY or Charles River Avian Vaccine Services, Norwich, CT) or

MDCK cells (NBL-2; ATCC® CCL-34™), as described (11). Stocks were harvested as pooled and clarified cell culture supernatant/lysate or egg allantoic/amniotic fluid and stored at -80°C. Stocks were titered by tissue culture infectious dose 50 percent ($TCID_{50}$) assay as described in standard protocols (16-19).

In vivo protection experiments

Mice were injected *i.p.* with 3-100 µg of rIgGs diluted to 200 µl in PBS. Three hours later, mice were anesthetized by *i.p.* injection of ketamine (85 mg/mL) and xylazine (13 mg/mL) and infected intranasally by either ≥10×LD_{50} (5×10^4 PFU; the LD_{50} was determined to be between 1.5×10^3 PFU and 5×10^3 PFU) of B/Malaysia/2506/2004, or 3×LD_{50} (1.5×10^4 PFU) or 10×LD_{50} (5×10^4 PFU) of A/Aichi/2/1968 X-31 (H3N2) in 40 µL volumes. X-31 typically was used at a lower dose than B/Malaysia due to the former's greater virulence, demonstrated by more rapid weight loss after infection. Mice were monitored daily for survival and body weight loss until 13 days post-challenge. The humane endpoint was set at 20% body weight loss relative to the initial body weight at the time of infection.

Influenza microneutralization assay

Virus neutralization endpoint titers were determined using the influenza microneutralization assay on Madin-Darby Canine Kidney (MDCK) cells (London Line, FR-58) as described (9, 16-19). Monoclonal Abs were diluted to 100 µg/ml in assay diluent and then serially two-fold diluted. Neutralizing antisera served as neutralization controls. Diluted Abs were mixed with 100 $TCID_{50}$ of influenza virus, dispensed to microtiter plates, and incubated for 60 min at 37°C, 5% CO_2. MDCK cells (1.5×10^4) were added to each well, then incubated overnight at 37°C, 5% CO_2. The cells were washed once in PBS, fixed with pre-chilled (−20°C) 80% acetone and incubated at RT for 10 min, then air-dried. After washing the fixed cells, primary antibody (mouse anti-influenza A nucleoprotein, Millipore MAB8251; or mouse anti-influenza B nucleoprotein, Millipore MAB8661) diluted 1:4,000 was incubated with the cells for 60 min at RT, followed by extensive washing. Secondary antibody (HRP-conjugated goat anti-mouse IgG; KPL 474-1802; 1:4,000) was incubated with cells for 60 min at RT, followed by extensive washing and HRP substrate development at RT. The HRP reaction was stopped with 0.5 N sulfuric acid. Absorbance was measured at 490 nM wavelength in a Synergy H1 automated microplate reader (BioTek Instruments). Wells with absorbance values ≤50% of virus-only control wells were scored as neutralization positive. Data were expressed as the mean of the reciprocal of the final dilution that was positive for neutralization. All samples were assayed in duplicate.

Reagents obtained through BEI Resources, NIAID, NIH include: influenza A virus A/Aichi/2/1968 (H3N2), NR-3177; polyclonal influenza virus A/Aichi/2/1968 (H3N2) serum (guinea pig), NR-3126. Influenza A virus A/Solomon Islands/3/2006 (H1N1; FR-331) and MDCK London cells (FR-58) were obtained through the International Reagent Resource (formerly the Influenza Reagent Resource), Influenza Division, WHO Collaborating Center for Surveillance, Epidemiology and Control of Influenza, Centers for Disease Control and Prevention, Atlanta, GA, USA.

Cryo-EM sample preparation

B/MY04 EHA2 was incubated with 3-fold molar excess (per EHA2 polypeptide chain) of S1V2-72 Fab at 4°C for 60 min. Fab:EHA2 complex was isolated by size-exclusion chromatography over a Superdex 200 Increase 10/300 GL column (GE Healthcare) equilibrated

in PBS. Pooled fractions containing the Fab:EHA2 complex were concentrated to 3 mg/ml. 3.0 µL of sample was deposited onto 300 mesh Quantifoil Au 0.6/1.0 grids that had been glow discharged in a PELCO easiGLOW (Ted Pella) at 0.39 mBar, 15 mA for 30 s. Samples were vitrified in 100% liquid ethane using a Vitrobot Mark IV (Thermo Fisher Scientific), with a wait time of 10 s, blot time of 8 s and a blot force of 6 at 100% humidity.

Cryo-EM data collection and processing

Cryo-EM data were recorded on a 300 kV Titan Krios G3i Microscope (Thermo Fisher Scientific) equipped with a K3 direct electron detector (Gatan) and a GIF quantum energy filter (20 eV; Gatan) at the Harvard Cryo-Electron Microscopy Center for Structural Biology at Harvard Medical School. Data were acquired in counting mode, using image shift and real-time coma correction by beamtilt with the automated data collection software SerialEM (20); nine holes were visited per stage position, acquiring one movie per hole. Details of the data collection and dataset parameters are summarized in Supporting Table 2. Dose-fractionated images were gain normalized, aligned, dose-weighted and summed using MotionCor2 (21). Patch contrast transfer function (CTF) and defocus value estimation were performed using CryoSPARC (22). Details of the data processing strategy are shown in Supp. Fig. 3. In short, particle picking in a subset of micrographs was carried out using CryoSPARC, followed by 2D classification and culling of junk classes within CryoSPARC. Selected 2D classes were used as templates to pick particles from a larger subset of micrographs, which, after 2D classification, were culled to 609,447 particles. Ab-initio reconstruction within CryoSPARC yielded an initial model (from 246,000 particles) that was used to create 2D templates for another round of particle picking. This time, particles were picked from all 14,273 micrographs; these particles were culled by multiple rounds of 2D classification to select the best-looking classes of particles appearing to include 2 Fabs bound to an EHA2 rod (282,599 particles). After Ab-Initio reconstruction and heterogeneous refinement of five 3D classes from these particles, the 135,398 particles in the three best-looking classes were used for another round of ab-initio reconstruction with three 3D classes. The largest and best-looking 3D class (59,527 particles) was subjected to homogeneous refinement and local refinement within CryoSPARC to produce the final ~5Å reconstruction. UCSF ChimeraX (23, 24) was used to fit the AF2-predicted models of EHA2 and S1V2-72 to cryo-EM map. Structural biology applications used in this project were compiled and configured by SBGrid (25).

STATISTICAL ANALYSES

All statistical analyses were performed using GraphPad Prism (v10) software.

In vivo challenge. Statistical significance of differences in mouse survival after lethal influenza challenge was calculated by the log-rank (Mantel-Cox) test, with Bonferroni's correction for multiple comparisons applied.

Flow cytometry and serological analyses in EHA2-immunized mice. Data were log-transformed to achieve normal distribution, then statistically significant differences between groups were detected by Welch's version of one-way ANOVA. Where ANOVA detected significant differences in means ($P<0.05$), the multiplicity-adjusted P-values of Dunnett's T3 post-test were reported with asterisks on the corresponding graphs. *, $P<0.05$; **, $P<0.01$; ***, $P<0.001$.

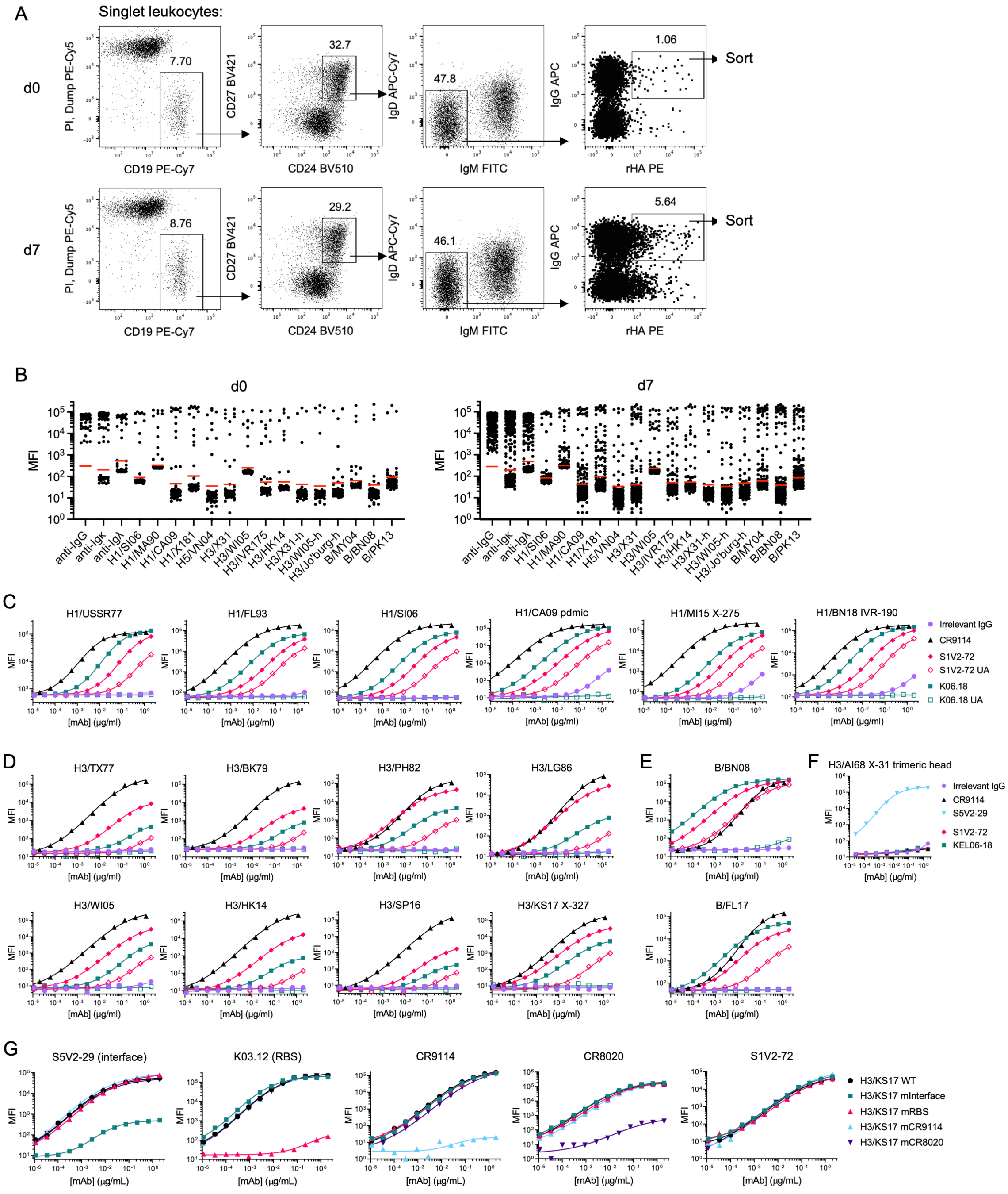

Fig. S1. Identification and characterization of broadly reactive HA mAbs. A) Example flow cytometry gating strategy for isolation of HA-binding IgG+ Bmem from human donor S1 before (d0) or one week after (d7) immunization with seasonal influenza vaccine. B) MFI values of Nojima culture supernatant IgGs binding to antigen beads in a Luminex assay. Each symbol represents clonal IgG from a single Bmem cell. Horizontal red lines denote the binding threshold (mean plus 6 SD of signal from control wells containing no B cells). C-G) Luminex assay results depicting rIgG binding to full-length soluble ectodomain (FLsE) of prefusion H1 HA (C), FLsE prefusion H3 HA (D), FLsE prefusion B HA (E), H3/X31 trimeric head (i.e., lacking the stem domain; F), or FLsE prefusion wildtype (WT) and mutant forms of H3/KS17 (G). The mutants, mInterface, mRBS, mCR9114, and mCR8020, have clusters of point mutations designed (28) to eliminate binding of head interface, receptor binding site, CR9114-like, and CR8020-like Abs, respectively.

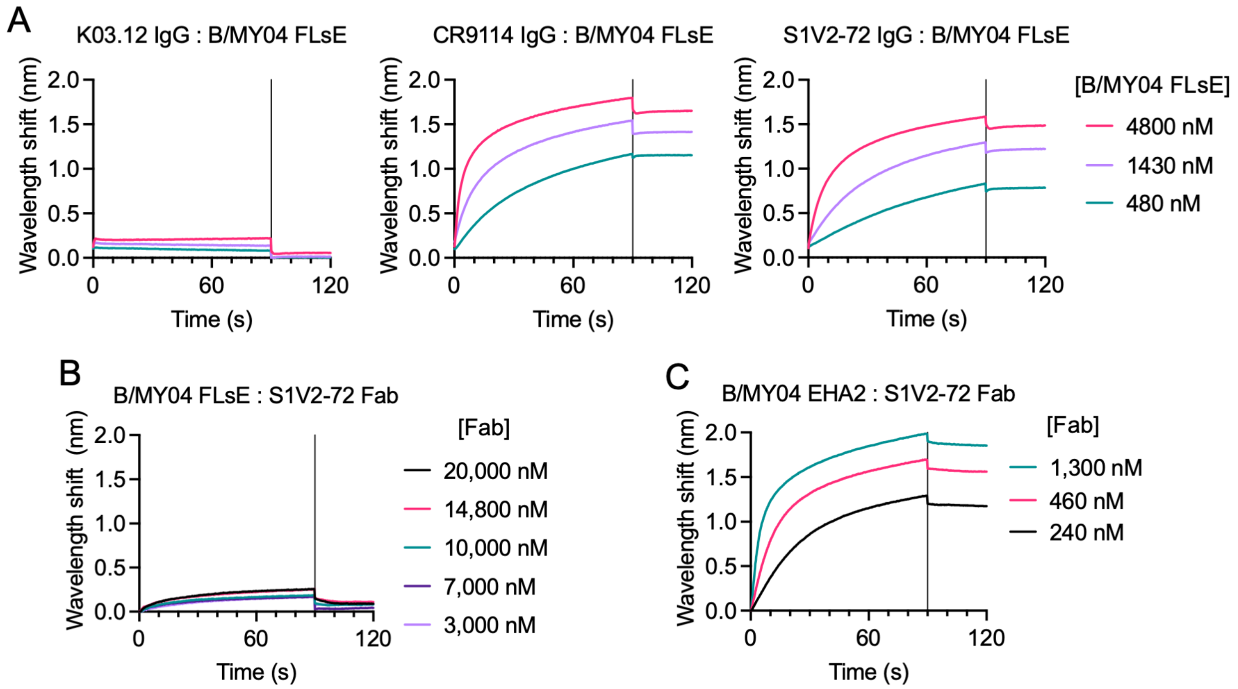

Fig. S2. S1V2-72 binds postfusion HA more avidly than prefusion HA. A) BLI traces showing the association of B/MY04 FLsE HA0 trimers with IgG-loaded BLI sensors. The IAV-specific IgG K03.12 (11) served as a negative control for HA binding. B-C) BLI traces showing the association of S1V2-72 Fab with B/MY04 FLsE-loaded (B) or B/MY04 EHA2-loaded (C) BLI sensors. In all traces, protein binding to the loaded sensor was measured as wavelength shift ($\Delta\lambda$), in nm.

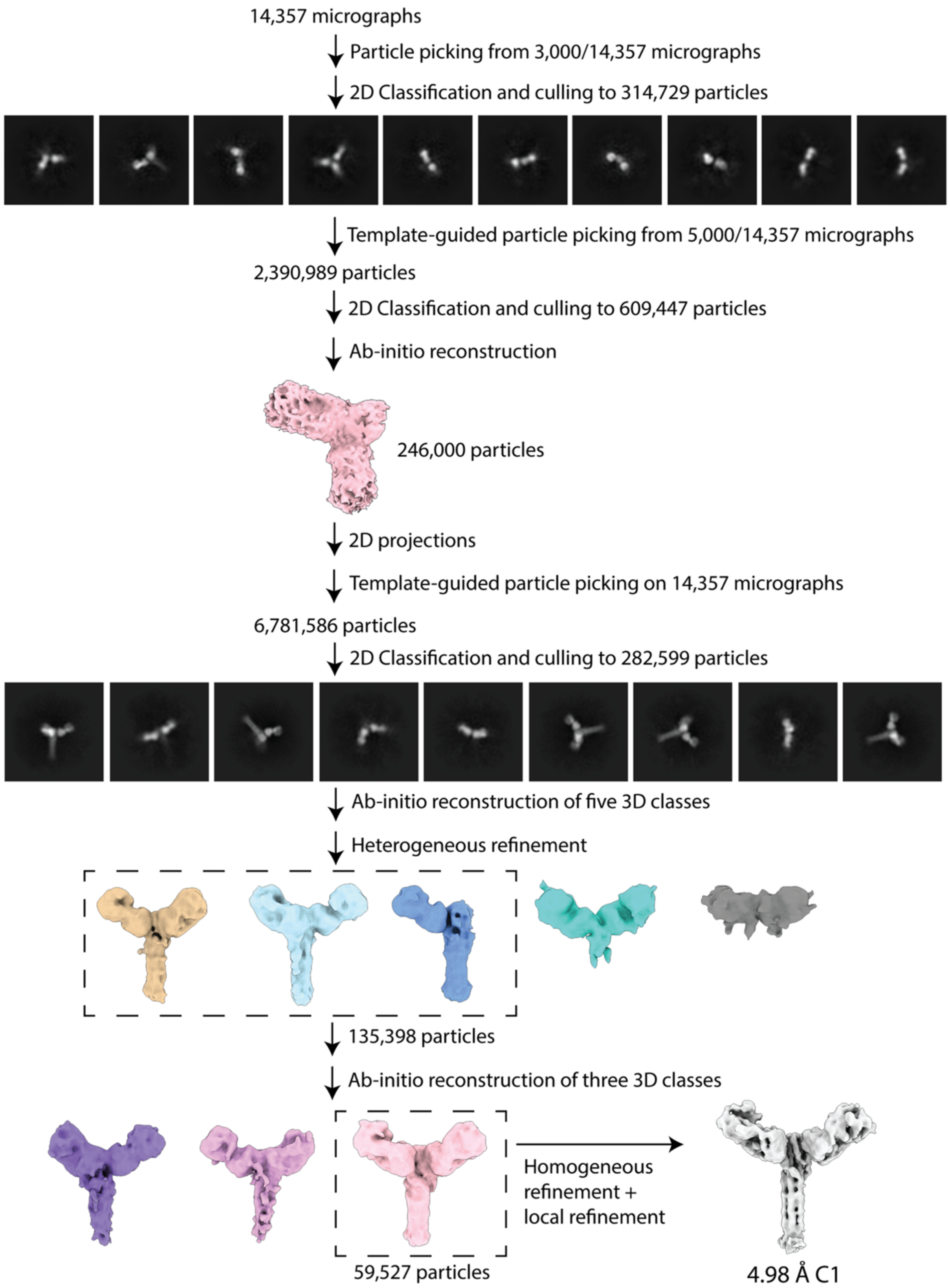

Fig. S3. Schematic depicting the strategy for processing cryo-EM data.

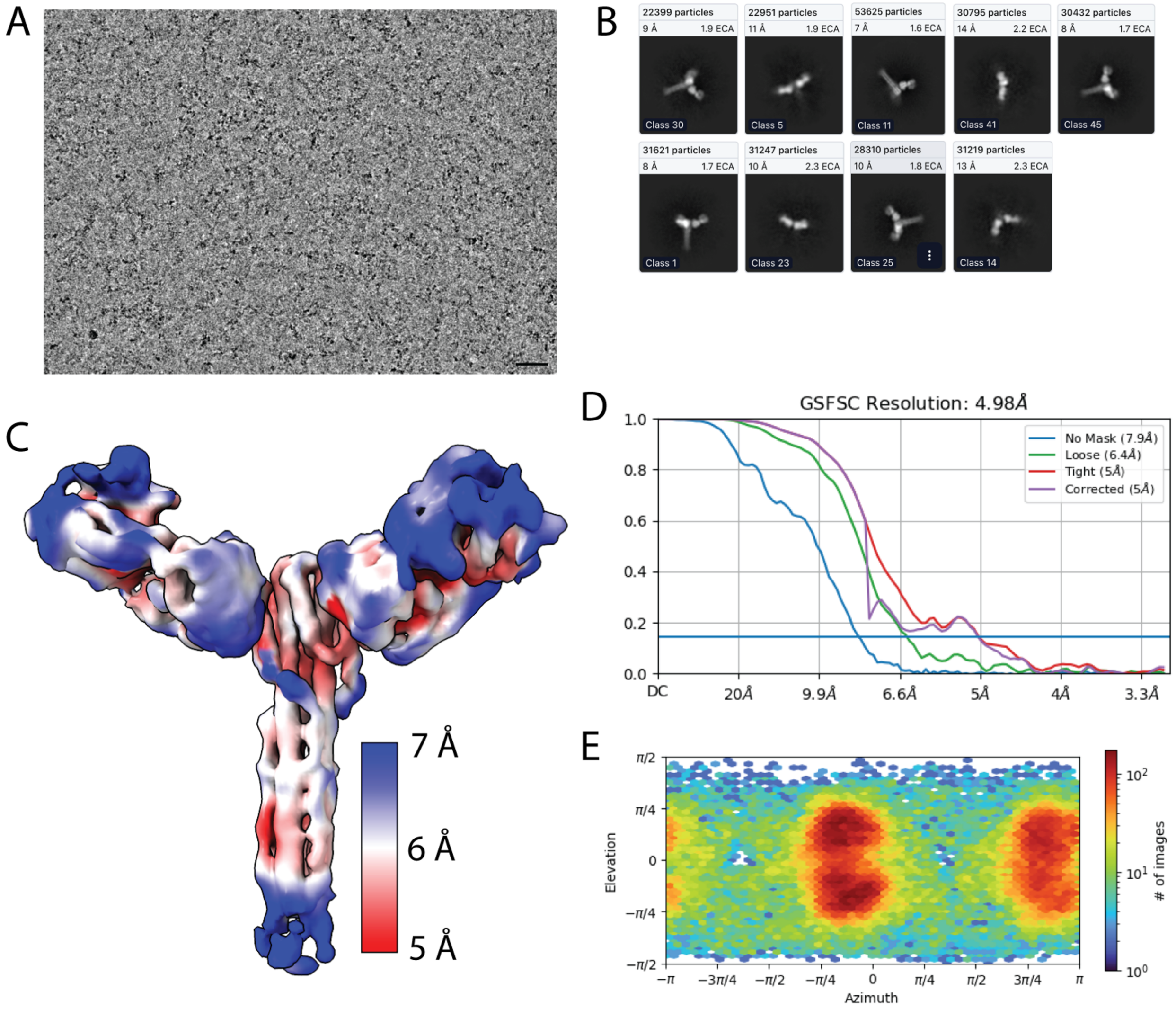

Fig. S4. A) Representative micrograph of S1V2-72 Fab bound to B/MY04 EHA2, embedded in vitreous ice (scale bar = 300 Å), low pass filtered for clarity. B) Selected 2D class averages of S1V2-72 Fab:EHA2 complexes. C) Reconstruction of two S1V2-72 Fabs bound to B/MY04 EHA2, filtered and colored by local resolution. D) Gold-standard Fourier shell correlation (GSFSC) curves from CryoSPARC. E) Viewing direction distribution plot.

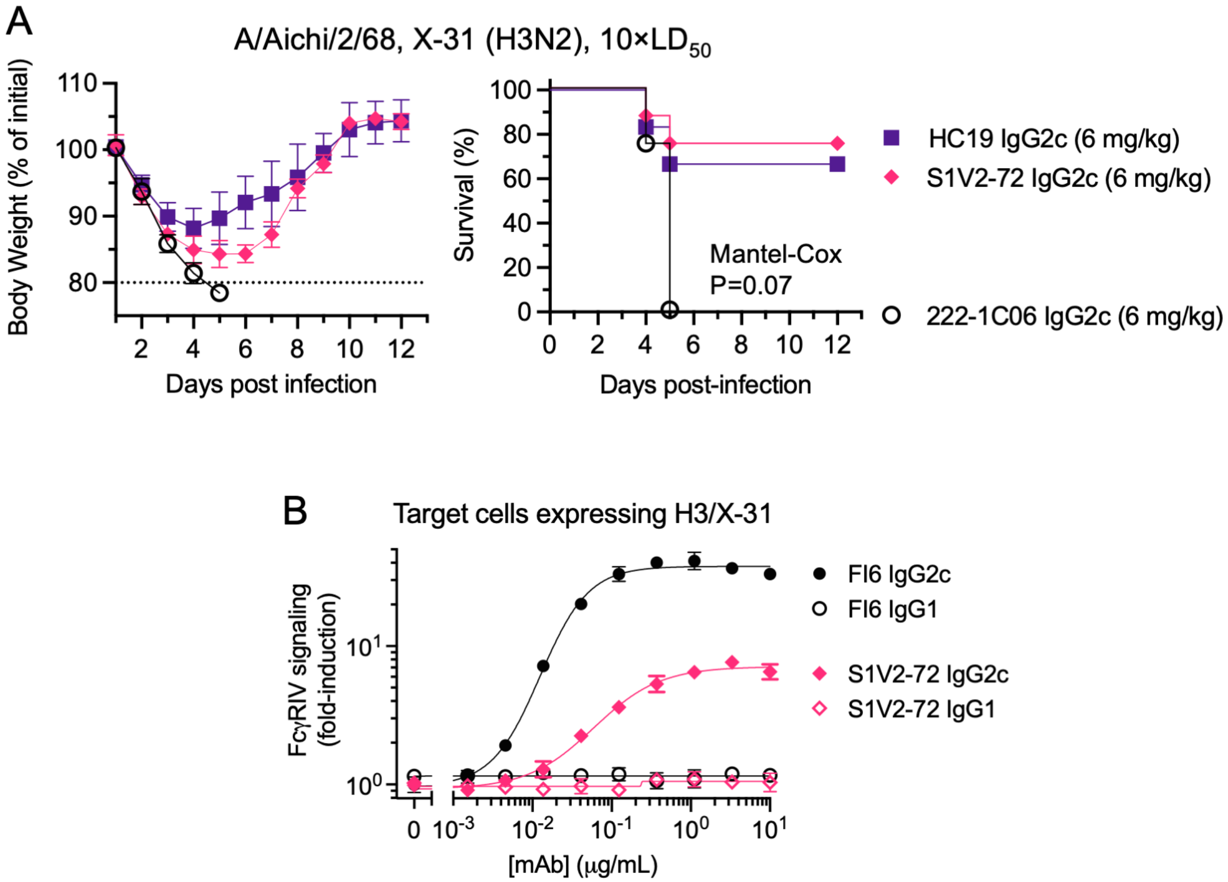

Fig. S5. S1V2-72 protects mice from lethal infection with a high dose of IAV. Mice were injected with mAbs and subsequently infected with H3N2 IAV. Post-infection weight loss (mean ± SEM) and survival were tracked over time. B) Results of an *in vitro* ADCC proxy assay. Mouse FcγRIV-expressing effector cells and HA-expressing target cells were co-cultured in the presence of serially diluted mouse IgG2c or mouse IgG1 mAbs. FcγRIV activation was measured as luminescence output (mean ± S.D.).

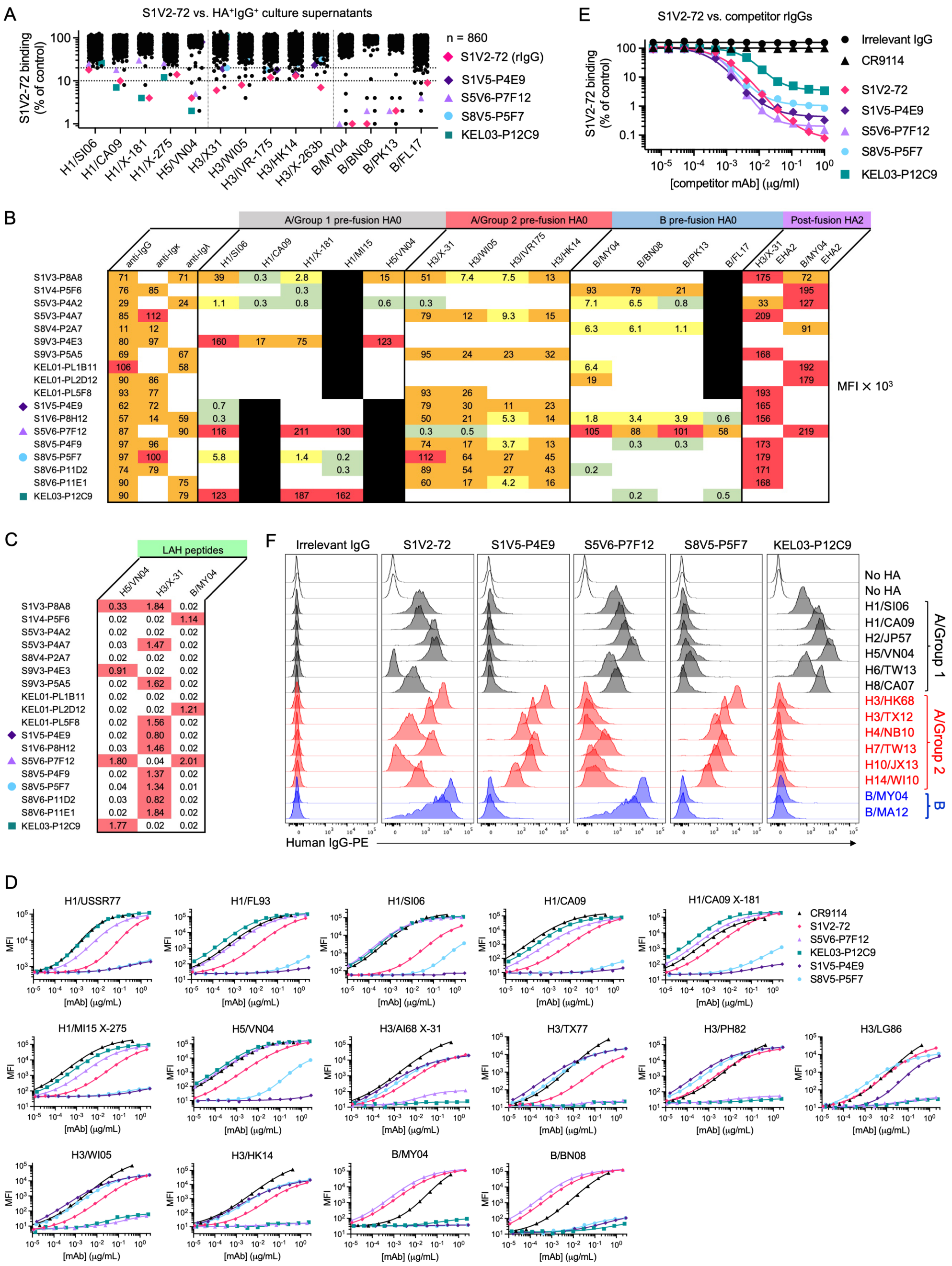

Fig. S6. Characterization of human mAbs that compete with S1V2-72 for HA binding. A) Inhibition of S1V2-72 binding to HAs by Nojima culture supernatant IgGs. Samples selected for further characterization as rIgGs are marked with unique colored symbols. Dotted horizontal lines denote 80% and 90% inhibition. B) Luminex MFI values for Nojima culture supernatant IgGs binding to antigen beads. Results are shown for the IgGs that inhibited S1V2-72 binding by ≥90%, and color-coded as in Fig. 1A, except that blank white cells denote values below the limit of detection, and green cells denote MFI values <1000. Black cells were not measured. C) ELISA OD values for culture supernatant IgGs binding to LAH peptides. Values above background are highlighted red. D) Luminex assay results for rIgG binding to HA-conjugated beads. E) Results of a Luminex competitive inhibition assay. Each curve depicts S1V2-72 binding to HA in the presence of serially diluted competitor rIgGs. HAs used were B/MY04 EHA2 (for irrelevant IgG, CR9114, S1V2-72, S5V6-P7F12), H3/X31 EHA2 (for S1V5-P4E9, S8V5-P5F7), or H5/VN04 FLsE (for KEL03-P12C9). F) Flow cytometry histograms depicting recombinant IgG binding to K530 cell lines expressing recombinant, native HA on the cell surface.

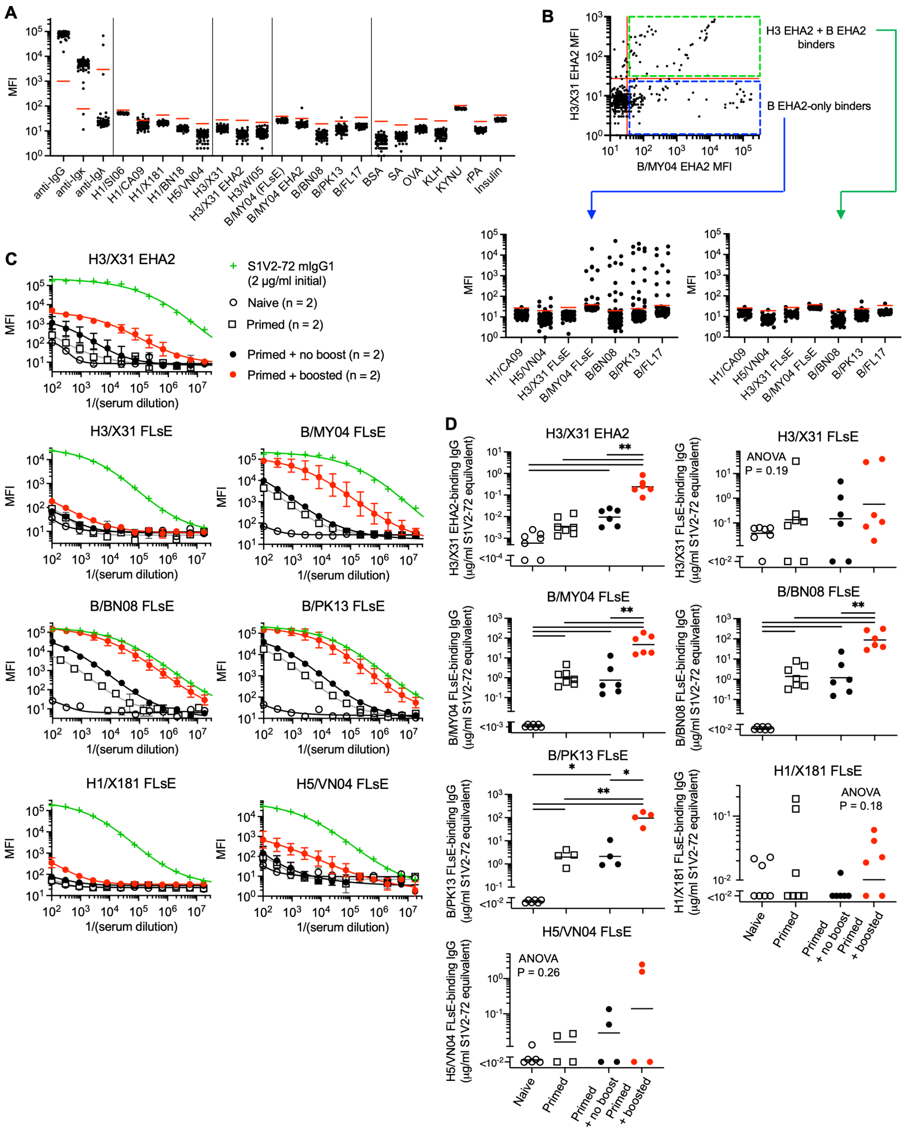

Fig. S7. B/MY04 EHA2 vaccination elicits potent GC and serum Ab responses against the S1V2-72 epitope. A) Luminex MFI values for culture supernatant IgG binding. Each symbol represents clonal IgG from a single mature follicular B cell. Horizontal red lines denote the binding threshold (mean plus 6 SD of signal from control wells containing no B cells). Data are from one mouse; they are representative of three independent experiments with three mice total. B) Top: Luminex MFI values for culture supernatant IgGs from GC B cells. Bottom left: Luminex MFI values for supernatant IgGs that bind B/MY04 EHA2, but not H3/X31 EHA2. Bottom right: Luminex MFI values for IgGs that bind both B/MY04 EHA2 and H3/X31 EHA2. Each symbol represents a clonal IgG from a single B cell isolated 18 d post-prime with B/MY04 EHA2. Data were pooled from 3 mice from one independent experiment, and are representative of two independent experiments with 5 mice total. Solid red lines denote the binding thresholds, as in (A). C) Luminex MFI values for HA binding by serially diluted serum IgG from mice treated as in Fig 5A. Each curve depicts the geometric mean ± SEM. Binding by recombinant S1V2-72 mouse IgG1 standard (2 μg/ml initial, then serially 3-fold diluted) is shown for comparison. Data are representative of three independent experiments. D) Concentrations of HA-binding serum IgG, normalized to S1V2-72 mouse IgG1 standard Ab (as shown in [C]). Each symbol represents one mouse. Data were pooled from 2-3 independent experiments with 2-3 animals per group per experiment. Horizontal lines depict geometric means. Asterisks (C, D) denote statistically significant differences at P<0.05 (*), P<0.01 (**).

Table S1. Summary of new[a] human Nojima culture screening results

Donor ID	No. wells screened	No. IgG[+b]	No. rHA[+c]
KEL01	96	79	13
KEL03	384	336	84
S1	1,572	1,139	221
S5	1,152	880	87
S8	1,788	1,310	208
S9	338	221	34
S12	1,116	765	80
**Total**	**6,446**	**4,730**	**727**

Notes:
[a]Additional (n = 915) HA-binding culture supernatant IgGs used in this study have been reported already (11, 15).
[b]Number of IgG-positive wells, determined by Luminex binding assay.
[c]Number of HA-binding IgGs, determined by Luminex binding assay.

Table S2: Cryo-EM data collection and processing statistics

**Data collection and processing**	
Magnification	105,000
Voltage (kV)	300
Electron exposure ($e^-/Å^{-2}$)	76.12
Defocus range (μm)	0.8, 2.2
Pixel size (Å)	0.825
Symmetry imposed	C1
Initial particle images (no.)	6,781,586
Final particle images (no.)	59,527
Map Resolution (Å)	4.98
FSC threshold	0.143
Map resolution range (Å)	5.0 - 7.0

Table S3. Summary of mouse Nojima culture screening results

	Experiment 1		Experiment 2		Total	
	Day 18 GC B	MF B[a]	Day 18 GC B	MF B[a]	Day 18 GC B	MF B[a]
IgG^+ / total screened[b]	546/4,032	73/96	736/4,032	78/96	1,282/8,064	151/192
Cloning efficiency (%)	14	76	18	81	16	79
Total B/MY04 $EHA2^+$ cultures[c]	172/546	1/76[d]	182/736	6/78[d]	354/1,282	7/151[d]
B/MY04 $EHA2^+$ + B/MY04 $FLsE^+$ crossreactive cultures[e]	12/172	0/1	49/182	0/6	61/354	0/7
B/MY04 $EHA2^+$ + B/BN08 $FLsE^+$ crossreactive cultures[e]	16/172	0/1	53/182	0/6	69/354	0/7
B/MY04 $EHA2^+$ + B/PK13 $FLsE^+$ crossreactive cultures[e]	20/172	0/1	65/182	0/6	85/354	0/7
B/MY04 $EHA2^+$ + H3/X31 $EHA2^+$ crossreactive cultures[e]	46/172	0/1	49/182	0/6	95/354	0/7
B/MY04 $EHA2^+$ + Group 1 HA^+ crossreactive cultures[f]	9/172	0/1	11/182	0/6	20/354	0/7

Notes:
[a]Resting mature follicular B cells
[b]Number of IgG-positive samples divided by the number of samples screened.
[c]Number of all B/MY04 $EHA2^+$ samples divided by the number of IgG^+ samples.
[d]The samples bound B/MY04 EHA2 just above the limit of detection.
[e]Number of samples that crossreacted with B/MY04 EHA2 and another antigen, divided by the total number of B/MY04 EHA2-reactive samples.
[f]Number of samples that crossreacted with B/MY04 EHA2 and H1/X181 FLsE and/or H5/VN04 FLsE, divided by the total number of B/MY04 EHA2-reactive samples.

SI References

1. X. M. Luo *et al.*, Engineering human hematopoietic stem/progenitor cells to produce a broadly neutralizing anti-HIV antibody after in vitro maturation to human B lymphocytes. *Blood* **113**, 1422-1431 (2009).
2. K. Y. Su, A. Watanabe, C. H. Yeh, G. Kelsoe, M. Kuraoka, Efficient Culture of Human Naive and Memory B Cells for Use as APCs. *J Immunol* **197**, 4163-4176 (2016).
3. M. Kuraoka *et al.*, Complex Antigens Drive Permissive Clonal Selection in Germinal Centers. *Immunity* **44**, 542-552 (2016).
4. S. Song *et al.*, A cell-based multiplex immunoassay platform using fluorescent protein-barcoded reporter cell lines. *Commun Biol* **4**, 1338 (2021).
5. J. R. Whittle *et al.*, Broadly neutralizing human antibody that recognizes the receptor-binding pocket of influenza virus hemagglutinin. *Proc Natl Acad Sci U S A* **108**, 14216-14221 (2011).
6. H. Xu *et al.*, Key mutations stabilize antigen-binding conformation during affinity maturation of a broadly neutralizing influenza antibody lineage. *Proteins* **83**, 771-780 (2015).
7. A. G. Schmidt *et al.*, Preconfiguration of the antigen-binding site during affinity maturation of a broadly neutralizing influenza virus antibody. *Proc Natl Acad Sci U S A* **110**, 264-269 (2013).
8. D. D. Raymond *et al.*, Influenza immunization elicits antibodies specific for an egg-adapted vaccine strain. *Nat Med* **22**, 1465-1469 (2016).
9. K. R. McCarthy *et al.*, Differential immune imprinting by influenza virus vaccination and infection in nonhuman primates. *Proc Natl Acad Sci U S A* **118** (2021).
10. J. Chen, J. J. Skehel, D. C. Wiley, N- and C-terminal residues combine in the fusion-pH influenza hemagglutinin HA(2) subunit to form an N cap that terminates the triple-stranded coiled coil. *Proc Natl Acad Sci U S A* **96**, 8967-8972 (1999).
11. K. R. McCarthy *et al.*, Memory B Cells that Cross-React with Group 1 and Group 2 Influenza A Viruses Are Abundant in Adult Human Repertoires. *Immunity* **48**, 174-184 e179 (2018).
12. M. Kuraoka *et al.*, Infant Antibody Repertoires during the First Two Years of Influenza Vaccination. *mBio* **13**, e0254622 (2022).
13. T. B. Kepler, Reconstructing a B-cell clonal lineage. I. Statistical inference of unobserved ancestors. *F1000Res* **2**, 103 (2013).
14. X. Brochet, M. P. Lefranc, V. Giudicelli, IMGT/V-QUEST: the highly customized and integrated system for IG and TR standardized V-J and V-D-J sequence analysis. *Nucleic Acids Res* **36**, W503-508 (2008).
15. A. Watanabe *et al.*, Antibodies to a Conserved Influenza Head Interface Epitope Protect by an IgG Subtype-Dependent Mechanism. *Cell* **177**, 1124-1135 e1116 (2019).
16. CDC, Influenza virus microneutralization assay. (Immunology and Pathogenesis Branch, Influenza Division, CDC US Department of Health and Human Services). (2007).
17. CDC, Influenza Virus Microneutralization Assay H1N1 Pandemic Response. (Centers for Disease Control US Department of Health and Human Services). (2009).
18. WHO, Serological diagnosis of influenza by microneutralization assay. (World Health Organization). (2010).
19. WHO, Manual for the laboratory diagnosis and virological surveillance of inflluenza. (World Health Organization). (2011).
20. D. N. Mastronarde, Automated electron microscope tomography using robust prediction of specimen movements. *J Struct Biol* **152**, 36-51 (2005).
21. S. Q. Zheng *et al.*, MotionCor2: anisotropic correction of beam-induced motion for improved cryo-electron microscopy. *Nat Methods* **14**, 331-332 (2017).
22. A. Punjani, J. L. Rubinstein, D. J. Fleet, M. A. Brubaker, cryoSPARC: algorithms for rapid unsupervised cryo-EM structure determination. *Nat Methods* **14**, 290-296 (2017).
23. T. D. Goddard *et al.*, UCSF ChimeraX: Meeting modern challenges in visualization and analysis. *Protein Sci* **27**, 14-25 (2018).
24. E. F. Pettersen *et al.*, UCSF ChimeraX: Structure visualization for researchers, educators, and developers. *Protein Sci* **30**, 70-82 (2021).
25. A. Morin *et al.*, Collaboration gets the most out of software. *Elife* **2**, e01456 (2013).
